# Supplementary material for: The Effectiveness of Tobacco Dependence Education in Health Professional Students’ Practice: A Systematic Review and Meta-Analysis of Randomized Controlled Trials
Source: Int J Environ Res Public Health. 2019 Oct 28;16(21):4158. doi: 10.3390/ijerph16214158 (PMC6862178; doi:10.3390/ijerph16214158)
Supplement: Supplementary file 1 [file ijerph-16-04158-s001.pdf]

Supplementary Section S1. Search strategy

PubMed at https://www.ncbi.nlm.nih.gov/pubmed/

Searches done: July 28, 2010 (n=567); June 11, 2012 (n=90); May 21, 2014 (n=56); April 6, 2016 (n=84); November 25, 2017 (n=27)

Search	Query
#1 - #5	("Tobacco Use Cessation"[Mesh] OR "smoking cessation") AND ("Teaching"[Mesh] OR "Students, Health Occupations"[Mesh] OR "Schools, Health Occupations"[Mesh] OR "Preceptorship"[Mesh] OR "Faculty"[Mesh] OR "Professional Competence"[Mesh] OR "Education, Pharmacy"[Mesh] OR "Education, Nursing"[Mesh] OR "Education, Medical"[Mesh] OR "Education, Dental"[Mesh] OR "Health Education, Dental"[Mesh] OR "Curriculum"[Mesh])
Date Limit: January 1 1990 – November 25, 2017. No language limits	

CINAHL EBSCOhost through Brandon University (https://www.brandonu.ca/library/databases/#E)

Searches done on August 11, 2011 (n=601); June 15, 2012 (n=149); May 15, 2014 (n=93); April 6, 2016 (n=77); November 25, 2017 (n=46)

Search	Query
#1- #5	((MH "Smoking Cessation Programs") OR (MH "Smoking Cessation")) AND ((MH "Curriculum+") OR (MH "Education, Clinical+") OR (MH "Dental Health Education") OR (MH "Education, Allied Health+") OR (MH "Education, Associate+") OR (MH "Education, Chiropractic") OR (MH "Education, Dental") OR (MH "Education, Medical+") OR (MH "Education, Midwifery") OR (MH "Education, Nursing+") OR (MH "Education, Pharmacy") OR (MH "Internship and Residency") OR (MH "Professional Competence+") OR (MH "Faculty, Health Occupations+") OR (MH "Learning Methods+") OR (MH "Outcomes of Education") OR (MH "Colleges and Universities+") OR (MH "Student Attitudes") OR

	(MH "Student Knowledge") OR (MH "Student Performance Appraisal+") OR (MH "Students, Health Occupations+") OR (MH "Students, Pre-Nursing") OR (MH "Students, Undergraduate") OR (MH "Teaching+"))
Date Limit: January 1990 – November 25, 2017 Limit to Peer Reviewed. No language limits	

EMBASE Database: Ovid at http://www.ovid.com/site/index.jsp

Searched on July 21, 2012 (n=932); June 29, 2014 (n=167); April 18, 2016 (n=169); December 6, 2017 (n=93)

Search	Query
#1 - #4	All search terms found through Mapping tool and searched as 'Focused' (that is why * appears before search terms)<br>**(***smoking cessation/ or *smoking cessation program/)<br>AND<br>(*education/ or *academic achievement/ or *curriculum/ or *curriculum development/ or *education program/ or *educational model/ or *health education/ or *"outcome of education"/ or *physical education/ or *social work education/ or *teaching/ or *medical education/ or *clinical education/ or *clinical supervision/ or *dental education/ or *medical school/ or *residency education/ or *surgical training/ or *paramedical education/ or *allied health education/ or *chiropractic education/ or *midwifery education/ or *nurse training/ or *occupational therapy education/ or *physical therapy education/ or *nursing education/ or *nurse midwifery education/ or *school/ or *college/ or *medical school/ or *university/ or *paramedical personnel/ or *chiropractor/ or *dental assistant/ or *dentist/ or *health practitioner/ or *occupational therapist/ or *pharmacist/ or *physiotherapist/ or *respiratory therapist/ or *speech language pathologist/ or *midwife/ or *nurse midwife/ or *nurse/ or *advanced practice nurse/ or *nursing student/ or *baccalaureate nursing

	student/ or *graduate nursing student/ or *male nursing student/ or *paramedical student/ or *chiropractic student/ or *dental hygiene student/ or *dental student/ or *occupational therapy student/ or *pharmacy student/ or *physical therapy student/ or *respiratory therapy student/ or *medical personnel/ or *medical specialist/ or *resident/ or *medical student/ or *physician/ or *anesthetist/ or *cardiologist/ or *dermatologist/ or *emergency physician/ or *endocrinologist/ or *general practitioner/ or *gerontologist/ or *gynecologist/ or *internist/ or *nephrologist/ or *orthopedic specialist/ or *pediatrician/ or *psychiatrist/ or *radiologist/ or *surgeon/ or *urologist/)
Date Limit: 1990 – 2017. No language limits	

Scopus Database: Elsevier at https://www.elsevier.com/solutions/scopus

Searched on July 31, 2012 (n=1320); June 5, 2014 (n=203); April 18, 2016 (n=166); December 6, 2017 (n=136)

Search	Query
#1 - #4	((TITLE-ABS-KEY("Tobacco Use Cessation" OR "smoking cessation") **AND** TITLE-ABS-KEY(education OR curriculum OR teaching OR "Professional Competence" OR "Learning Methods" OR training OR "Program Evaluation" OR schools OR internship OR residency OR students OR faculty)) AND PUBYEAR > 1989) **AND** ((TITLE-ABS-KEY("Health Occupations" OR "Health Personnel" OR "Allied Health" OR pharmacy OR pharmacist OR nursing OR nurses OR medical OR dental OR dentist OR dental hygiene OR doctors OR physicians OR psychologists OR chiropractors OR psychiatry OR psychiatrists) OR TITLE-ABS-KEY(chiropractors OR psychiatry OR psychiatrists OR midwifery OR midwives OR "Mental Health Workers" OR "Occupational Therapists" OR "Physical Therapists" OR "Speech Therapists" OR

	"respiratory therapists" OR optometrists OR "social workers")) AND PUBYEAR > 1989)
Date Limit: 1990 – 2017. No language limits	

Web of Science Database: Thompson Reuters at https://www.brandonu.ca/library/databases/#W

Searched on July 25, 2012 (n=996); May 21, 2014 (n=231); April 13, 2016 (n=231); November 25, 2017 (n=178)

Search	Query
#1 - #4	("Tobacco Use Cessation" or "smoking cessation") AND (Education OR Curriculum OR Teaching OR "Professional Competence" OR "Learning Methods" OR Training OR "Program Evaluation" OR Schools OR Internship OR Residency OR Students OR Faculty) AND ("Health Occupations" OR "Health Personnel" OR "Allied Health" OR Pharmacy OR Pharmacist OR Nursing OR Nurses OR Medical OR Dental OR Dentist OR Dental hygiene OR Doctors OR Physicians OR Psychologists OR Chiropractors OR Psychiatry OR Psychiatrists OR Midwifery OR Midwives OR "Mental Health Workers" OR "Occupational Therapists" OR "Physical Therapists" OR "Speech Therapists" OR "respiratory therapists" OR Optometrists OR "social workers")
Date Limit: 1990 – 2017. No language limits	

Academic Search Premier Database: EBSCOhost at Brandon University (https://www.brandonu.ca/library/databases/#E)

Searched on Sept 7, 2010 (n=418); July 15, 2012 (n=107); May 16, 2014 (n=137); April 6, 2016 (n=143); November 25, 2017 (n=136)

Search	Query

#1 - #5	(DE "smoking cessation" OR DE "SMOKING cessation programs") AND (DE "NURSES" OR DE "MALE nurses" OR DE "NURSE practitioners" OR DE "Psychiatric Nurses" OR DE "Health Personnel" OR DE "MEDICAL personnel" OR DE "ALLIED health personnel" OR DE "Occupational Therapists" OR DE "Physical Therapists" OR DE "Speech Therapists" OR DE "CHIROPRACTORS" OR DE "OPTOMETRISTS" OR DE "PHARMACISTS" OR DE "PHYSICIANS" OR DE "General Practitioners" OR DE "Gynecologists" OR DE "Neurologists" OR DE "Obstetricians" OR DE "Pediatricians" OR DE "Surgeon" OR DE "Mental Health Personnel" OR DE "School Psychologists" OR DE "Clinical Psychologists" OR DE "Psychiatrists" OR DE "Dentists" OR DE "Dental hygiene" OR DE "Internists" OR DE "MEDICINE" OR DE "Naturopathic medicine" OR DE "DENTISTRY" OR DE "Psychology" OR DE "GYNECOLOGY" OR DE "MIDWIFERY" OR DE "NURSING" OR DE "OBSTETRICS" OR DE "PEDIATRICS" OR DE "PHARMACY" OR DE "PSYCHIATRY" OR DE "Medical schools" OR DE "Medical education" OR DE "Medical students" OR DE "Dental schools" OR DE "Dental education" OR DE "Dental students" OR DE "Nursing education" OR DE "Nursing schools" OR DE "Nursing students" OR DE "social workers" OR DE "SOCIAL work education" OR DE "Curriculum" OR DE "Curricula" OR DE "Ophthalmologists" OR DE "Respiratory Therapy")
Date Limit: January 1990 – November 2017; Scholarly (Peer Reviewed) Journals. No language limits	

PsychINFO Database: EBSCOhost at Brandon University (https://www.brandonu.ca/library/databases/#E)

Searched on July 25, 2011 (n=614); June 25, 2012 (n=69); May 15, 2014 (n=74); April 6, 2016 (n=75); November 25, 2017 (n=39)

Search	Query
#1 - #5	DE "Smoking Cessation" AND (DE "Health Personnel" OR DE "Allied Health Personnel" OR DE "Medical Personnel" OR DE "Mental Health Personnel" OR DE "Occupational Therapists" OR DE "Physical Therapists" OR DE "Speech Therapists" OR DE "Dentists" OR DE "Military Medical Personnel" OR DE "Nurses" OR DE "Optometrists" OR DE "Pharmacists" OR DE "Physical Therapists" OR DE "Physicians" OR DE "Psychiatric Hospital Staff" OR DE "Clinical Psychologists" OR DE "Psychiatric Hospital Staff" OR DE "Psychiatric Nurses" OR DE "Psychiatric Social Workers" OR DE "Psychiatrists" OR DE "School Psychologists" OR DE "Curriculum" OR DE "Curriculum Development" OR DE "Health Education" OR DE "Psychology Education" OR DE "Graduate Psychology Education" OR DE "Higher Education" OR DE "Graduate Education" OR DE "Postgraduate Training" OR DE "Undergraduate Education" OR DE "Dental Education" OR DE "Graduate Psychology Education" OR DE "Medical Education" OR DE "Rehabilitation Education" OR "Clinical Psychology Graduate Training" OR DE "Clinical Psychology Internship" OR DE "Medical Internship" OR DE "Medical Residency" OR DE "Psychiatric Training" OR DE "Nursing Education" OR DE "Psychiatric Nurses" OR DE "Public Health Service Nurses" OR DE "School Nurses" OR DE "Family Physicians" OR DE "General Practitioners" OR DE "Gynecologists" OR DE "Internists" OR DE "Neurologists" OR DE "Obstetricians" OR DE "Pediatricians" OR DE "Psychiatrists" OR DE "Surgeon" OR DE "College Students" OR DE "Community College Students" OR DE "Nursing Students" OR DE

	"Dental Students" OR DE "Graduate Students" OR DE "Medical Students" OR DE "Postgraduate Students")
Date Limit: January 1990 – November 2017; Scholarly (Peer Reviewed) Journals. No language limits	

Health Source Nursing – **Academic** Database: EBSCOhost at Brandon University (https://www.brandonu.ca/library/databases/#E)

Searched on August 16, 2012 (n=351); May 16, 2014 (n=61); April 6, 2016 (n=68); November 25, 2017 (n=44)

Search	Query
#1 - #4	("SMOKING cessation" OR "Tobacco cessation") AND (DE "Education" OR DE "CURRICULA (Courses of study)" OR DE "CURRICULA (Courses of study) -- Aims & objectives" OR DE "COURSE content (Education)" OR DE "CURRICULUM change" OR DE "CURRICULUM enrichment" OR DE "CURRICULUM frameworks" OR DE "CURRICULUM implementation" OR DE "CURRICULUM planning" OR DE "CURRICULUM research" OR DE "CURRICULUM theories" OR DE "CURRICULUM-based assessment" OR DE "EDUCATIONAL programs" OR DE "EDUCATIONAL standards" OR DE "PROGRAM validation (Education)" OR DE "EDUCATIONAL outcomes" OR DE "EDUCATIONAL benefits" OR DE "HEALTH education" OR DE "OUTCOME assessment (Education)" OR DE "PHYSICAL education" OR DE "SOCIAL work education" OR DE "PREMEDICAL education" OR DE "CLINICAL medical education" OR DE "MEDICAL education" OR DE "MEDICINE -- Study & teaching" OR DE "MEDICINE -- Study & teaching (Graduate)" OR DE "MEDICINE -- Study & teaching (Preceptorship)" OR DE "FAMILY medicine -- Study & teaching" OR DE "PARAMEDICAL education" OR DE "ALLIED health personnel" OR DE "CHIROPRACTIC -- Study & teaching" OR DE "PHYSICAL therapy -- Study & teaching" OR DE "NURSING -- Study &

	teaching" OR DE "NURSES -- In-service training" OR DE "NURSING -- Study & teaching (Associate degree)" OR DE "NURSING -- Study & teaching (Graduate)" OR DE "NURSING -- Study & teaching (Preceptorship)" OR DE "UNIVERSITIES & colleges -- Curricula" OR DE "PUBLIC health nursing -- Study & teaching" OR DE "CHIROPRACTORS" OR DE "DENTAL auxiliary personnel" OR DE "DENTISTRY -- Study & teaching" OR DE "DENTAL personnel" OR DE "HEALTH occupations schools" OR DE "HEALTH occupations students" OR DE "HEALTH practitioners" OR DE "MENTAL health personnel" OR DE "OCCUPATIONAL therapists" OR DE "PHARMACISTS" OR DE "PHYSICAL therapists" OR DE "RESPIRATORY therapists" OR DE "SPEECH therapists" OR DE "MIDWIVES" OR DE "NURSES" OR DE "NURSING students" OR DE "DENTAL hygienists" OR DE "DENTAL students" OR DE "PHARMACY -- Study & teaching" OR DE "PHARMACY students" OR DE "MEDICAL personnel" OR DE "MEDICAL research personnel" OR DE "MEDICAL teaching personnel" OR DE "MEDICAL students" OR DE "PHYSICIANS" OR DE "ANESTHESIOLOGISTS" OR DE "CARDIOLOGISTS" OR DE "DERMATOLOGISTS" OR DE "EMERGENCY physicians" OR DE "ENDOCRINOLOGISTS" OR DE "PHYSICIANS (General practice)" OR DE "GERONTOLOGISTS" OR DE "GYNECOLOGISTS" OR DE "INTERNISTS" OR DE "NEPHROLOGISTS" OR DE "ORTHOPEDISTS" OR DE "PEDIATRICIANS" OR DE "PSYCHIATRISTS" OR DE "RADIOLOGISTS" OR DE "SURGEONS" OR DE "UROLOGISTS" OR DE "ANESTHESIOLOGY -- Study & teaching" OR DE "PSYCHIATRY -- Study & teaching" OR DE "EMERGENCY medical personnel" OR DE "OPERATING room personnel" OR DE "ACUPUNCTURISTS" OR DE "HEALTH care teams -- Training of" OR DE "PUBLIC health personnel -- Education" DE "PUBLIC health personnel" OR DE "OBSTETRICS -- Study & teaching" OR DE "PHYSICIANS' assistants -- Education" OR

	DE "OPTOMETRISTS" OR DE "HOMEOPATHIC physicians" OR DE "OBSTETRICIANS" OR DE "OPHTHALMOLOGISTS" OR DE "GYNECOLOGY -- Study & teaching" OR DE "NURSING -- Research -- Study & teaching" OR DE "OPHTHALMOLOGY -- Study & teaching" OR DE "OPTOMETRY -- Study & teaching" OR DE "PSYCHOLOGY -- Study & teaching" OR DE "PSYCHOLOGY -- Study & teaching (Graduate)" OR DE "PHARMACOLOGY -- Study & teaching" OR DE "PHYSIOLOGY -- Study & teaching" OR DE "PSYCHIATRY -- Study & teaching (Residency)" OR DE "SURGERY -- Study & teaching" OR DE "SOCIAL work education" OR DE "CHIROPRACTIC schools" OR DE "DENTAL schools" OR DE "MEDICAL schools" OR DE "NATUROPATHIC schools" OR DE "NURSING schools" OR DE "OPTOMETRY schools" OR DE "PHARMACY colleges" OR DE "PHYSICAL therapy schools" OR DE "PUBLIC health schools" OR DE "ONCOLOGY -- Study & teaching" OR DE "NEUROLOGY -- Study & teaching" OR DE "NEUROLOGISTS" OR DE"ONCOLOGISTS")
Date Limit: January 1990 – November 2017; Scholarly (Peer Reviewed) Journals. No language limits	

Google Scholar Google at https://scholar.google.ca/

Searched on Sept 5, 2012 (n=354); May 21, 2014 (n=51); April 13, 2016 (n=89); November 25, 2017 (n=74)

Search	Query
#1 - #4	All in title: Education OR Curriculum OR curricula OR Teaching OR Learning OR Training OR Program OR Schools OR school OR Residency OR Students OR student AND ("smoking cessation" OR "tobacco cessation" OR "tobacco use intervention") -"high school"

Limited to: All in title. Decided to limit to title words only since results sets are otherwise too large. No other way to limit in a meaningful way. Date Limit: 1990-2017. Unchecked citations and patents. No language limits

AMED Database: Ovid at http://www.ovid.com/site/index.jsp

Searched on September 1, 2012 (n=75); July 31, 2014 (n=10); April 26, 2016 (n=9); December 6, 2017 (n=1)

Search	Query
#1 - #4	("Tobacco Use Cessation" or "smoking cessation")<br>AND<br>(Education OR Curriculum OR Teaching OR "Professional Competence" OR "Learning Methods" OR Training OR "Program Evaluation" OR Schools OR Internship OR Residency OR Students OR Faculty)<br>OR<br>("Health Occupations" OR "Health Personnel" OR "Allied Health" OR Pharmacy OR Pharmacist OR Nursing OR Nurses OR Medical OR Dental OR Dentist OR Dental hygiene OR Doctors OR Physicians OR Psychologists OR Chiropractors OR Psychiatry OR Psychiatrists OR Midwifery OR Midwives OR "Mental Health Workers" OR "Occupational Therapists" OR "Physical Therapists" OR "Speech Therapists" OR "respiratory therapists" OR Optometrists OR "social workers")
Date Limit: 1990 – 2017. Searches as all Keywords (uncheck map to subject terms). No language limits	

TRIP Online http://www.tripdatabase.com/

Searched on Sep 17, 2012 (n=280); June 16, 2014 (n=91); April 26, 2016 (n=443); December 6, 2017 (n=244)

Search	Query
#1 - #4	("smoking cessation" OR "Tobacco Use Cessation" OR "Tobacco Cessation") AND (Education OR Curriculum OR Teaching OR "Professional Competence" OR "Learning Methods" OR Training OR "Program Evaluation" OR Schools OR Internship OR Residency OR Students OR Faculty) AND ("Health Occupations" OR "Health Personnel" OR "Allied Health" OR Pharmacy OR Pharmacist OR Nursing OR Nurses OR Medical OR Dental OR Dentist OR Dental hygiene OR Doctors OR Physicians OR Psychologists OR Chiropractors OR Psychiatry OR Psychiatrists OR Midwifery OR Midwives OR "Mental Health Workers" OR "Occupational Therapists" OR "Physical Therapists" OR "Speech Therapists" OR "respiratory therapists" OR Optometrists OR "social workers")
Date Limit: 1990 – 2017 Limited to: Key primary research OR primary research. No language limits	

ERIC Database: EBSCOhost at Brandon University (https://www.brandonu.ca/library/databases/#E)

Searched on August 12, 2011 (n=65); June 20, 2012 (n=13); May 16, 2014 (n=6); April 6, 2016 (n=7) November 25, 2017 (n=1)

Search	Query
#1 - #4	DE "smoking" AND DE ("Medical Education" OR "Graduate Medical Education" OR "Nursing Education" OR "Pharmaceutical Education" OR "Allied Health Occupations Education" OR "Nursing Students" OR

	"Medical Students" OR "Medical Schools" OR "Dental Schools" OR "Allied Health Occupations" OR "Health Personnel" OR "Allied Health Personnel" OR "Mental Health Workers" OR "Nurses" OR "Physicians" OR "Psychologists" OR "Dentistry" OR "Pharmacy" OR "Nursing" OR "Obstetrics" OR "Pediatrics" OR "Psychiatry")
Date Limit: January 1990 – November 2017 Limited to: Peer reviewed. No language limits	

Natural Standard (https://naturalmedicines.therapeuticresearch.com/databases.aspx)
Search conducted in Sept 02, 2012; August 12, 2014; and May 2 and May 8, 2016; January 7, 2018 (n=0)

Search	Query
#1 - #4	1. "smoking cessation" – reference databases<br>2. “Tobacco”<br>In 2012 we discovered 9 records, 2 of which had bibliographies that were of interest. These bibliographies where then checked.<br>Bibliography 1 – 263 references – we kept 2 – both records are duplicates in other databases.<br>Bibliography 2 – 228 references – we kept 0.<br>No results for 2014, 2016 or 2018 searches.
Date Limit: 2012, 2014, 2016, 2018. No language limit. In 2016 the journal website was changed to www.naturalmedicines.com with a paid subscription. In 2016 and 2018 searched Health and Wellness, then Medical Conditions with Addictions and Smoking Cessation or Tobacco	

Education Research Complete EBSCOhost at Brandon University (https://www.brandonu.ca/library/databases/#E)
Searched on August 21, 2012 (n=204); July 8, 2014 (n=38); April 15, 2016 (n=17); December 6, 2017 (n=19)

Search	Query
#1 - #4	DE "Smoking Cessation"<br>AND<br>(DE "NURSES" OR DE "MALE nurses" OR DE "NURSE practitioners" OR DE "Psychiatric Nurses" OR DE "Health Personnel" OR DE "MEDICAL personnel" OR DE "ALLIED health personnel" OR DE "Occupational Therapists" OR DE "Physical Therapists" OR DE "Speech Therapists" OR DE "CHIROPRACTORS" OR DE "OPTOMETRISTS" OR DE "PHARMACISTS" OR DE "PHYSICIANS" OR DE "General Practitioners" OR DE "Gynecologists" OR DE "Neurologists" OR DE "Obstetricians" OR DE "Pediatricians" OR DE "Surgeon" OR DE "Mental Health Personnel" OR DE "School Psychologists" OR DE "Clinical Psychologists" OR DE "Psychiatrists" OR DE "Dentists" OR DE "Dental hygiene" OR DE "Internists" OR DE "MEDICINE" OR DE "Naturopathic medicine" OR DE "DENTISTRY" OR DE "Psychology" OR DE "GYNECOLOGY" OR DE "MIDWIFERY" OR DE "NURSING" OR DE "OBSTETRICS" OR DE "PEDIATRICS" OR DE "PHARMACY" OR DE "PSYCHIATRY" OR DE "Medical schools" OR DE "Medical education" OR DE "Medical students" OR DE "Dental schools" OR DE "Dental education" OR DE "Dental students" OR DE "Nursing education" OR DE "Nursing schools" OR DE "Nursing students" OR DE "social workers" OR DE "SOCIAL work education" OR DE "Curriculum" OR DE "Curricula" OR DE "Ophthalmologists" OR DE "Respiratory Therapy")<br>AND<br>(DE "Education" OR DE "CURRICULA (Courses of study)" OR DE "CURRICULA (Courses of study) -- Aims & objectives" OR DE "COURSE content (Education)" OR DE "CURRICULUM change" OR DE "CURRICULUM enrichment" OR DE "CURRICULUM frameworks" OR DE "CURRICULUM implementation" OR DE "CURRICULUM planning" OR DE "CURRICULUM research" OR DE "CURRICULUM

	theories" OR DE "CURRICULUM-based assessment" OR DE "EDUCATIONAL programs" OR DE "EDUCATIONAL standards" OR DE "PROGRAM validation (Education)" OR DE "EDUCATIONAL outcomes" OR DE "EDUCATIONAL benefits" OR DE "HEALTH education" OR DE "OUTCOME assessment (Education)" OR DE "PHYSICAL education" OR DE "SOCIAL work education" OR DE "PREMEDICAL education" OR DE "CLINICAL medical education" OR DE "MEDICAL education" OR DE "MEDICINE -- Study & teaching" OR DE "MEDICINE -- Study & teaching (Graduate)" OR DE "MEDICINE -- Study & teaching (Preceptorship)" OR DE "FAMILY medicine -- Study & teaching" OR DE "PARAMEDICAL education" OR DE "ALLIED health personnel" OR DE "CHIROPRACTIC -- Study & teaching" OR DE "PHYSICAL therapy -- Study & teaching" OR DE "NURSING -- Study & teaching" OR DE "NURSES -- In-service training" OR DE "NURSING -- Study & teaching (Associate degree)" OR DE "NURSING -- Study & teaching (Graduate)" OR DE "NURSING -- Study & teaching (Preceptorship)" OR DE "UNIVERSITIES & colleges -- Curricula" OR DE "PUBLIC health nursing -- Study & teaching" OR DE "CHIROPRACTORS" OR DE "DENTAL auxiliary personnel" OR DE "DENTISTRY -- Study & teaching" OR DE "DENTAL personnel" OR DE "HEALTH occupations schools" OR DE "HEALTH occupations students" OR DE "HEALTH practitioners" OR DE "MENTAL health personnel" OR DE "OCCUPATIONAL therapists" OR DE "PHARMACISTS" OR DE "PHYSICAL therapists" OR DE "RESPIRATORY therapists" OR DE "SPEECH therapists" OR DE "MIDWIVES" OR DE "NURSES" OR DE "NURSING students" OR DE "DENTAL hygienists" OR DE "DENTAL students" OR DE "PHARMACY -- Study & teaching" OR DE "PHARMACY students" OR DE "MEDICAL personnel" OR DE "MEDICAL research personnel" OR DE "MEDICAL teaching personnel" OR DE "MEDICAL students" OR DE "PHYSICIANS" OR DE "ANESTHESIOLOGISTS" OR DE

	"CARDIOLOGISTS" OR DE "DERMATOLOGISTS" OR DE "EMERGENCY physicians" OR DE "ENDOCRINOLOGISTS" OR DE "PHYSICIANS (General practice)" OR DE "GERONTOLOGISTS" OR DE "GYNECOLOGISTS" OR DE "INTERNISTS" OR DE "NEPHROLOGISTS" OR DE "ORTHOPEDISTS" OR DE "PEDIATRICIANS" OR DE "PSYCHIATRISTS" OR DE "RADIOLOGISTS" OR DE "SURGEONS" OR DE "UROLOGISTS" OR DE "ANESTHESIOLOGY -- Study & teaching" OR DE "PSYCHIATRY -- Study & teaching" OR DE "EMERGENCY medical personnel" OR DE "OPERATING room personnel" OR DE "ACUPUNCTURISTS" OR DE "HEALTH care teams -- Training of" OR DE "PUBLIC health personnel -- Education" DE "PUBLIC health personnel" OR DE "OBSTETRICS -- Study & teaching" OR DE "PHYSICIANS' assistants -- Education" OR DE "OPTOMETRISTS" OR DE "HOMEOPATHIC physicians" OR DE "OBSTETRICIANS" OR DE "OPHTHALMOLOGISTS" OR DE "GYNECOLOGY -- Study & teaching" OR DE "NURSING -- Research -- Study & teaching" OR DE "OPHTHALMOLOGY -- Study & teaching" OR DE "OPTOMETRY -- Study & teaching" OR DE "PSYCHOLOGY -- Study & teaching" OR DE "PSYCHOLOGY -- Study & teaching (Graduate)" OR DE "PHARMACOLOGY -- Study & teaching" OR DE "PHYSIOLOGY -- Study & teaching" OR DE "PSYCHIATRY -- Study & teaching (Residency)" OR DE "SURGERY -- Study & teaching" OR DE "SOCIAL work education" OR DE "CHIROPRACTIC schools" OR DE "DENTAL schools" OR DE "MEDICAL schools" OR DE "NATUROPATHIC schools" OR DE "NURSING schools" OR DE "OPTOMETRY schools" OR DE "PHARMACY colleges" OR DE "PHYSICAL therapy schools" OR DE "PUBLIC health schools" OR DE "ONCOLOGY -- Study & teaching" OR DE "NEUROLOGY -- Study & teaching" OR DE "NEUROLOGISTS" OR DE"ONCOLOGISTS")
Date Limit: January 1990 – December 6, 2017 Scholarly (Peer Reviewed) Journals. No language limits	

SocIndex Database: EBSCOhost at Brandon University (https://www.brandonu.ca/library/databases/#E)

Searched on August 11, 2011 (n=83); June 26, 2012 (n=10); May 16, 2014 (n=3); April 6, 2016 (n=1); November 25, 2017 (n=2)

Search	Query
#1-#5	((MH "Smoking Cessation Programs") OR (MH "Smoking Cessation")) AND ((MH "Curriculum+") OR (MH "Education, Clinical+") OR (MH "Dental Health Education") OR (MH "Education, Allied Health+") OR (MH "Education, Associate+") OR (MH "Education, Chiropractic") OR (MH "Education, Dental") OR (MH "Education, Medical+") OR (MH "Education, Midwifery") OR (MH "Education, Nursing+") OR (MH "Education, Pharmacy") OR (MH "Internship and Residency") OR (MH "Professional Competence+") OR (MH "Faculty, Health Occupations+") OR (MH "Learning Methods+") OR (MH "Outcomes of Education") OR (MH "Colleges and Universities+") OR (MH "Student Attitudes") OR (MH "Student Knowledge") OR (MH "Student Performance Appraisal+") OR (MH "Students, Health Occupations+") OR (MH "Students, Pre-Nursing") OR (MH "Students, Undergraduate") OR (MH "Teaching+"))
Date Limit: January 1990 – November 2017 Scholarly (Peer Reviewed) Journals. No language limits	

WorldCat OCLC at https://www.oclc.org/en/worldcat.html

Searched on July 2012 (n=104); July 2, 2014 (n=9); April 26, 2016 (n=5); November 25, 2017 (n=4)

Search	Query
#1 - #4	Subject ("smoking cessation" OR "Tobacco Use Cessation") OR (Tobacco and Cessation) AND Keywords (kw: Education OR kw: Curriculum OR kw: Teaching OR kw: Professional w Competence OR kw: Learning w Methods OR kw: Training OR kw: Program w Evaluation OR kw: Schools

	OR kw: Internship OR kw: Residency OR kw: Students OR kw: Faculty) AND (kw: Health w Occupations OR kw: Health w Personnel OR kw: Allied w Health OR kw: Pharmacy OR kw: Pharmacist OR kw: Nursing OR kw: Nurses OR kw: Medical OR kw: Dental OR kw: Dentist OR (kw: Dental and kw: hygiene) OR kw: Doctors OR kw: Physicians OR kw: Psychologists OR kw: Chiropractors OR kw: Psychiatry OR kw: Psychiatrists OR kw: Midwifery OR kw: Midwives OR kw: Mental w Health w Workers OR kw: Occupational w Therapists OR kw: Physical w Therapists OR kw: Speech w Therapists OR kw: respiratory w therapists OR kw: Optometrists OR kw: social workers)
Date Limit: 1990-2017 Not juvenile books. No language limits	

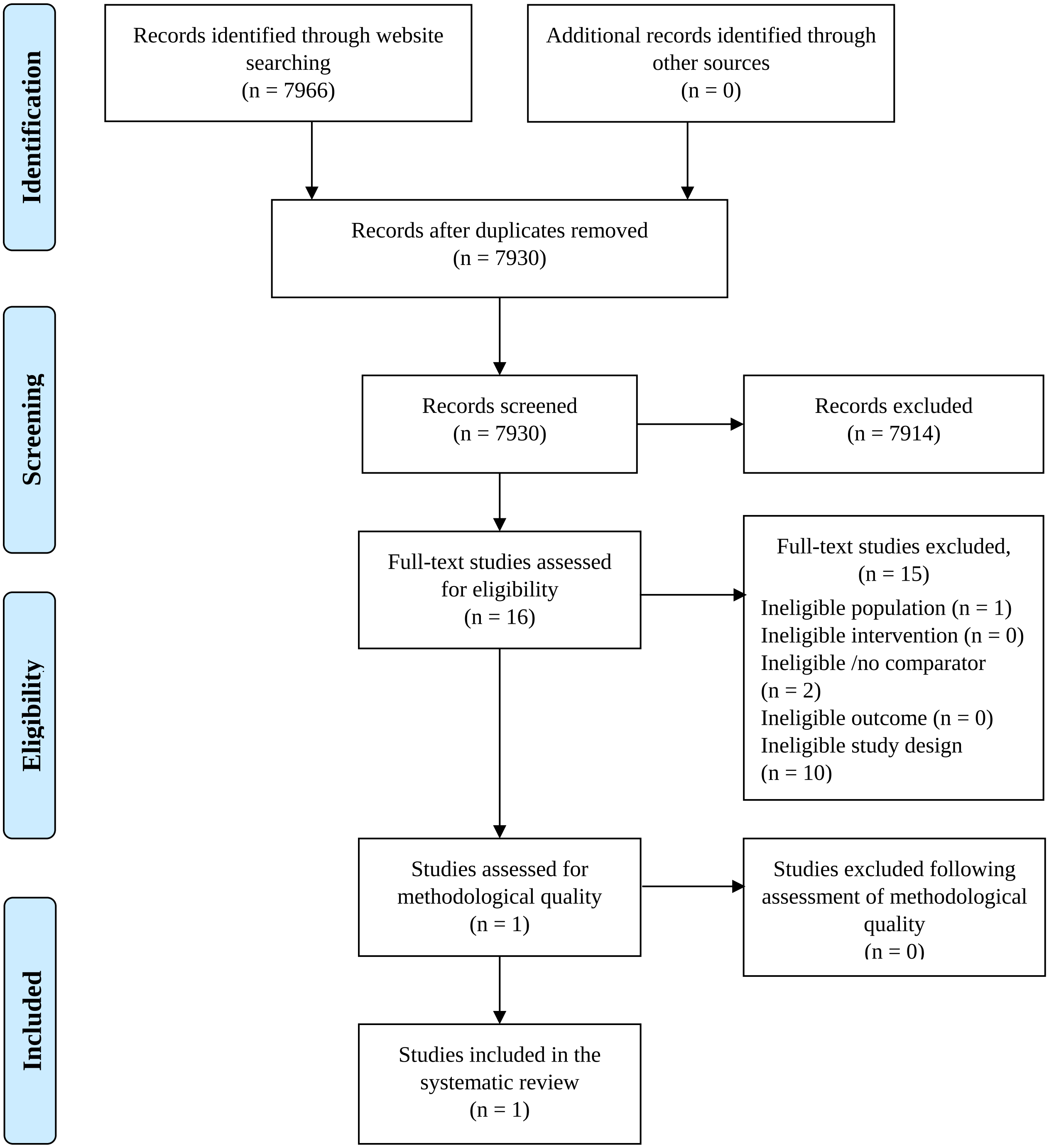

Supplementary Figure 1. PRISMA flow diagram of search results and study selection process for grey literature, 1990-2018. PRISMA = Preferred Reporting Items for Systematic Reviews and Meta-Analyses.

Supplementary Section S2. Published studies excluded on full text read

POPULATION

Sharifi M, Adams WG, Winickoff JP, Guo J, Reid M, Boynton-Jarrett R. Enhancing the electronic health record to increase counseling and quit-line referral for parents who smoke.

Reason for exclusion: This was a cohort study; the scores for residents and practising physicians were not separated.

Strayer SM, Rollins LK, Martindale JR. A handheld computer smoking intervention tool and its effects on physician smoking cessation counseling.

Reason for exclusion: The population included both medical faculty and residents, and their pre- and post-measures were reported together.

Uti O, Sofola O. Impact of an educational intervention on smoking counseling practice among Nigerian dentists and dental students.

Reason for exclusion: The population included practising dentists and dental students, and their scores were reported together.

INTERVENTION

Davies BL, Matte-Lewis L, O'Connor AM, Dulberg CS, Drake ER. Evaluation of the "Time to Quit" self-help smoking cessation program.

Reason for exclusion: In this study a cohort of students delivered first the control and then the experimental intervention to smoking subjects.

Kim SH, Lee JA, Kim KU, Cho HJ. Results of an inpatient smoking cessation program: 3-month cessation rate and predictors of success.

Reason for exclusion: The intervention was delivered to patients by medical residents and licensed nurses.

COMPARATOR

Brown RL, Pfeifer JM, Gjerde CL, Seibert CS, Haq CL. Teaching patient-centered tobacco intervention to first-year medical students.

Reason for exclusion: This study used a pre-test post-test design with one group of medical students. There was no comparison group.

Chan SS, So WK, Wong DC, Lam TH. Building an integrated model of tobacco control education in the nursing curriculum: findings of a students' survey.

Reason for exclusion: This cohort study used a pre-test post-test design. There was no control group.

Corelli RL, Kroon LA, Chung EP, Sakamoto LM, Gundersen B, Fenlon CM, et al. Statewide evaluation of a tobacco cessation curriculum for pharmacy students.

Reason for exclusion: This study used a cohort design with pre-test and post-test training surveys. There was no control group.

Cornuz J, Zellweger JP, Mounoud C, Decrey H, Pecoud A, Burnand B. Smoking cessation counseling by residents in an outpatient clinic.

Reason for exclusion: This was a cohort study with no control group.

Ehrenthal DB, Haynes SG, Martin KE, Hitch JA, Addo SF, O'Neill E, et al. Evaluation of the heart truth professional education campaign on provider knowledge of women and heart disease.

Reason for exclusion: This study used a pre-test post-test design without a control group.

Evans MW, Hawk C, Strasser SM. An educational campaign to increase chiropractic intern advising roles on patient smoking cessation.

Reason for exclusion: This was a cohort study and reported only post-test measures for chiropractic interns. There was no control group.

Fernandez K, Pandve HT, Debnath DJ. Use of interactive teaching methods in tobacco cessation program and examine it by using objective structured clinical exam.

Reason for exclusion: This was a cohort study with before and after measures, and no comparison group.

Franks AS. Enhancing team-based active learning through hands-on experience with nicotine replacement therapy.

Reason for exclusion: This study was a pre-test post-test course evaluation with no comparison group.

Geller AC, Prout MN, Sun T, Krane R, Schroy PC, Demierre MF, et al. Cancer skills laboratories for medical students: a promising approach for cancer education.

Reason for exclusion: This was a cohort study with no control group.

Gordon JS, Severson HH, Seeley JR, Christiansen S. Development and evaluation of an interactive tobacco cessation CD-ROM educational program for dental students.

Reason for exclusion: This study reported on a cohort using pre-test and post-test surveys to evaluate an educational strategy. There was no control group.

Henni A, Lee S, Kelly S, Zimmer J, Dehghani P, Lavoie AJ. Working together for primary prevention: Training medical students' to provide smoking cessation counseling.

Reason for exclusion: This study reported a pre-test post-test design. There was no comparison group.

Hudmon KS, Corelli RL, Chung E, Gundersen B, Kroon LA, Sakamoto LM, et al. Development and implementation of a tobacco cessation training program for students in the health professions.

Reason for exclusion: This study reported a cohort design, with no control group.

Hudmon KS, Kroon LA, Corelli RL, Saunders KC, Spitz MR, Bates TR, et al. Training future pharmacists at a minority educational institution: evaluation of the Rx for change tobacco cessation training program.

Reason for exclusion*:* This study was a cohort design with no control group.

Kelley FJ, Heath J, Crowell N. Using the Rx for Change tobacco curriculum in advanced practice nursing education.

Reason for exclusion: This was a pre-test post-test design and there was no control group.

Kosower E, Ernst A, Taub B, Berman N, Andrews J, Seidel J. Tobacco prevention education in a pediatric residency program.

Reason for exclusion: The study was a pre-test post-test design and there was no control group. Faculty members and resident scores were combined.

Leone FT, Evers-Casey S, Veloski J, Patkar AA, Kanzleiter L, et al. Short-, intermediate-, and long-term outcomes of Pennsylvania's continuum of tobacco education pilot project.

Reason for exclusion: This was a cohort study with before and after measures and no comparison group.

Miller PM, Heideman PW, Ravenel MC, Spangler JG, Mauldin MP, Hill EG, et al. Preliminary development and evaluation of online tobacco and alcohol modules for dental students.

Reason for exclusion: This was a cohort study with pre-test and post-test measures, and no control group.

Montner P, Bennett G, Brown C. An evaluation of a smoking cessation training program for medical residents in an inner-city hospital.

Reason for exclusion*:* This was a cohort study, using pre-test and post-test measures, and no control group.

Nieman LZ, Velasquez MM, Groff JY, Cheng L, Foxhall LE. Implementation of a smoking cessation counseling module in a preceptorship program.

Reason for exclusion: This study used a cohort design with pre-test and post-test measures. There was no comparison group.

Nieman LZ. A preclinical training model for chronic care education.

Reason for exclusion: This study was a cohort study with pre-test and post-test measures, and no control group.

O'Donnell JA, Hamilton MK, Markovic N, Close J. Overcoming barriers to tobacco cessation counselling in dental students.

Reason for exclusion: This study used a single cohort design and no control group.

Pati S. Putting tobacco cessation and prevention into undergraduate medical education.

Reason for exclusion: This study was a cohort study with pre-test and post-test measures, and no control group.

Prochaska JJ, Teherani A, Hauer KE. Medical students' use of the stages of change model in tobacco cessation counseling.

Reason for exclusion: This study was a cohort study with pre-test and post-test measures, and no control group.

Prochaska JJ, Fromont SC, Leek D, Hudmon KS, Louie AK, Jacobs MH, et al. Evaluation of an evidence-based tobacco treatment curriculum for psychiatry residency training programs.

Reason for exclusion: This study used a pre-test and post-test design with one cohort of medical residents across three sites. There was no comparison group.

Roman B, Borges N, Morrison AK. Teaching motivational interviewing skills to third-year psychiatry clerkship students.

Reason for exclusion: The study was a single cohort design with no control group.

Romito L, Schrader S, Zahl D. Using experiential learning and OSCEs to teach and assess tobacco dependence education with first-year dental students.

Reason for exclusion: This study used a cohort design over three consecutive years with pre-test and post-test measures, and no control group.

Saba M, Bittoun R, Saini B. A workshop on smoking cessation for pharmacy students.

Reason for exclusion: This study reported a cohort design, with pre-test and post-test measures, and no control group.

Saba M, Bittoun R, Saini B. Effect of a smoking cessation educational workshop on knowledge and attitudes in final year pharmacy students.

Reason for exclusion: This PowerPoint presentation reports on a cohort study with no control group.

Saito M, Nodate Y, Maruyama K, Tsuchiya M, Watanabe M, Niwa S. Establishment of a system for smoking cessation instruction practice using cognitive-behavioral therapy and a motivation interview method.

Reason for exclusion: This study was a cohort design, with pre-test and post-test measures, and no control group.

Scal P, Hennrikus D, Ehrlich L, Ireland M, Borowsky I. Preparing residents to counsel about smoking.

Reason for exclusion: This study used a pre-test and post-test design with one cohort and no control group.

Schmelz AN, Nixon B, McDaniel A, Hudmon KS, Zillich AJ. Evaluation of an online tobacco cessation course for health professions students.

Reason for exclusion: This study reported pre-test and post-training results from one student cohort and no control group.

Schwindt RG, McNelis AM, Sharp D. Evaluation of a theory-based education program to motivate nursing students to intervene with their seriously mentally ill clients who use tobacco.

Reason for exclusion: This study used a one group, pre-test post-test design, with no control group.

Shibly O. Effect of tobacco counseling by dental students on patient quitting rate.

Reason for exclusion: This study was a cohort study with pre-test and post-training measures, and no control group.

Shishani K, Stevens K, Dotson J, Riebe C. Improving nursing students' knowledge using online education and simulation to help smokers quit.

Reason for exclusion: This study was a cohort study with pre-test and post-test measures, and no control group.

Sohn M, Ahn Y, Park H, Lee M. Simulation-based smoking cessation intervention education for undergraduate nursing students.

Reason for exclusion: This study used one group with baseline and post-intervention measures, and no control group.

Quirk M, Ockene J, Kristeller J, Goldberg R, Donnelly G, Amick T, et al. Training family practice and internal medicine residents to counsel patients who smoke: Improvement and retention of counseling skills.

Reason for exclusion: This study used a cohort design with pre-test and post-test measures, and no control group.

Walsh SE, Singleton JA, Worth CT, Krugler J, Moore R, Wesley GC, et al. Tobacco cessation counseling training with standardized patients.

Reason for exclusion: This study used a cohort design with pre-program and post-program surveys, and no control group.

Whitehead D, Zucker SB, Stone J. Tobacco cessation education for advanced practice nurses.

Reason for exclusion: This cohort study used a pre-test post-test design, with no control group.

OUTCOME

Hymowitz N, Schwab J, Haddock CK, Burd KM, Pyle S. The pediatric residency training on tobacco project: baseline findings from the resident tobacco survey.

Reason for exclusion: This study reported only baseline scores on resident tobacco survey and counseling performance.

Hymowitz N, Schwab J, Haddock CK, Pyle S, Moore G, Meshberg S. The pediatric resident training on tobacco project: baseline findings from the Parent/Guardian tobacco survey.

Reason for exclusion: This study reports descriptive data on parents/guardians. No resident outcomes are reported.

Hymowitz N, Schwab J, Haddock CK, Pyle S, Meshberg S. The pediatric residency training on tobacco project: baseline findings from the patient tobacco survey.

Reason for exclusion: This study reported descriptive baseline data on patients who received residents' standard or special training intervention.

Hymowitz N, Schwab J, Haddock CK, Pyle S, Meshberg S. The pediatric resident training on tobacco project: interim findings.

Reason for exclusion: This study reports interim data on resident study outcomes.

Hymowitz N, Pyle SA, Haddock CK, Schwab JV. The pediatric residency training on tobacco project: four-year parent outcome findings.

Reason for exclusion: This study reported parents' reports of resident interventions. No resident outcome data included.

Lozano P, McPhillips HA, Hartzler B, Robertson AS, Runkle C, Scholz KA, et al. Randomized trial of teaching brief motivational interviewing to pediatric trainees to promote healthy behaviors in families.

Reason for exclusion: The focus is on motivational interviewing with asthma. Although it is a RCT, there is not enough focus on smoking cessation or prevention counseling. The outcomes relate to motivational interviewing skills in general, and are not consistent with required student outcomes in the current systematic review study.

Ockene JK, Adams A, Pbert L, Luippold R, Hebert JR, Quirk M, et al. The physician-delivered smoking intervention project - factors that determine how much the physician intervenes with smokers.

Reason for exclusion: The scores of the medical residents' outcomes cannot be identified.

Ridner SL, Ostapchuk M, Cloud RN, Myers J, Jorayeva A, Ling J. Using motivational interviewing for smoking cessation in primary care.

Reason for exclusion: This study reports patient outcomes. No resident educational outcomes are reported.

Schwindt RG, McNelis AM, Agley J. Curricular innovations in tobacco cessation education for prelicensure baccalaureate nursing students.

Reason for exclusion: This study reported self-perceived competence that might be similar to self-efficacy. No behavioral counseling skills or patient outcomes reported.

STUDY DESIGN

Allen SS, Bland CJ, Sawson SJ. A mini-workshop to train medical students to use a patient-centered approach to smoking cessation.

Reason for exclusion: This study is a cohort design with a comparison group.

Antal M, Forster A, Zalai Z, Barabas K, Spangler J, Braunitzer G, et al. A video feedback-based tobacco cessation counselling course for undergraduates-preliminary results.

Reason for exclusion: This study describes student satisfaction data from post-course evaluation.

Boehlecke B, Sperber AD, Kowlowitz V, Becker M, Contreras A, McGaghie WC. Smoking history-taking skills: a simple guide to teach medical students.

Reason for exclusion: This study reported an after-only non-experimental design and OSCEs were used post-intervention to assess smoking history-taking skills.

Boyd LD, Fun K, Madden TE. Initiating tobacco curricula in dental hygiene education: a descriptive report.

Reason for exclusion: This study reported descriptive survey results evaluating a revised curriculum.

Butler KM, Rayens MK, Zhang M, Maggio LG, Riker C, Hahn EJ. Tobacco dependence treatment education for baccalaureate nursing students.

Reason for exclusion: This study is a cohort design with two cohorts.

Campbell EE, Villagra VG, Rogers CS. Teaching and promoting smoking cessation counseling in primary care residencies: Description of a method.

Reason for exclusion: This study is a detailed description of a smoking cessation education intervention and student reactions to it.

Cato K, Hyun S, Bakken S. Response to a mobile health decision-support system for screening and management of tobacco use.

Reason for exclusion: This study was study was an observational design focussing on the experimental arm of a RCT.

Clareboets S, Sivarajasingam V, Chestnutt IG. Smoking cessation advice: knowledge, attitude and practice among clinical dental students.

Reason for exclusion: This study was a cross-sectional survey design.

Coan LL, Christen CA, Romito L. Evolution of a tobacco cessation curriculum for dental hygiene students at Indiana University School of Dentistry.

Reason for exclusion: This study was a cohort design. There were no pre-test measures and no comparison group.

Collins RL, D'Angelo S, Stearns SD, Campbell LR. Training pediatric residents to provide smoking cessation counseling to parents.

Reason for exclusion: This study's group assignments were made by clinic day and not randomized, which is open to confounders.

Dodge RAB, Cabana MD, O'Riordan MA, Heneghan A. What factors are important for pediatric residents' smoking cessation counseling of parents?

Reason for exclusion: This study was a cross-sectional survey design of two training programs.

Ehizele AO, Azodo CC, Ezeja EB, Ehigiator O. Nigerian dental students' compliance with the 4As approach to tobacco cessation.

Reason for exclusion: This study was a cross-sectional survey.

Eyler AE, Dicken LL, Fitzgerald JT, Oh MS, Wolf FM, Zweifler AJ. Teaching smoking-cessation counseling to medical students using simulated patients.

Reason for exclusion: This study reports a cohort design with post-intervention measures and no comparison group. The purpose was to evaluate student proficiency in counseling rather than to compare interventions.

Geller AC, Prout MN, Miller DR, Siegel B, Sun T, Ockene J, et al. Evaluation of a cancer prevention and detection curriculum for medical students.

Reason for exclusion: This study reported a design comparing student cohorts over four years.

Geller AC, Hayes RB, Leone F, Churchill LC, Leung K, Reed G, et al. Tobacco dependence treatment teaching by medical school clerkship preceptors: Survey responses from more than 1000 US medical students.

Reason for exclusion: This study was a cross-sectional survey.

Gelsky SC. Impact of a dental/dental hygiene tobacco-use cessation curriculum on practice.

Reason for exclusion: This study used a pre-audit and post-chart audit to evaluate a tobacco-use cessation program.

Giovino GA, Cummings KM, Koenigsberg MR, Sciandra RC. An evaluation of a physician training program on patient smoking cessation.

Reason for exclusion: This study reported a non-equivalent control group design. Participants were assigned to groups by non-random methods.

Grassi MC, Baraldo M, Chiamulera C, Culasso F, Raupach T, Ferketich AK, et al. Knowledge about health effects of cigarette smoking and quitting among Italian university students: the importance of teaching nicotine dependence and treatment in the medical curriculum.

Reason for exclusion: This study was a cohort study with a comparison group. Participant groups were created by non-random methods.

Hawk C, Kaeser MA, Beavers DV. Feasibility of using a standardized patient encounter for training chiropractic students in tobacco cessation counseling.

Reason for exclusion: This study was a description of an intervention to one cohort using post-intervention measures.

Hayes RB, Geller A, Churchill L, Jolicoeur D, Murray DM, Shoben A, et al. Teaching tobacco dependence treatment and counseling skills during medical school: rationale and design of the Medical Students helping patients Quit tobacco (MSQuit) group randomized controlled trial.

Reason for exclusion: This study reports a protocol for a proposed RCT.

Herold R, Schiekirka S, Brown J, Bobak A, McEwen A, Raupach T. Structured smoking cessation training for medical students: a prospective study.

Reason for exclusion: This study was a prospective cohort design with historical control. Groups were created in a non-random manner.

Humair J-P and Cornuz J. A new curriculum using active learning methods and standardized patients to train residents in smoking cessation.

Reason for exclusion: This study is a descriptive report of the educational content and methods of a proposed RCT.

Hymowitz N, Schwab J, Eckholdt H. Pediatric residency training on tobacco.

Reason for exclusion: This study was a controlled before and after study. The groups were created using non-random methods.

Klein JD, Portilla M, Goldstein A, Leininger L. Training pediatric residents to prevent tobacco use.

Reason for exclusion: This study was a controlled before and after study. The intervention and comparison groups were assigned in a non-random manner.

Kristina SA, Thavorncharoensap M, Pongcharoensuk P, Montakantikul P, Suansanae T, Prabandari YS. Effectiveness of tobacco education for pharmacy students in Indonesia.

Reason for exclusion: This study was a pre-test post-test cohort design with a comparison group.

Lee MT, Hishinuma ES, Derauf C, Guerrero AP, Iwaishi LK, Kasuya RT. Smoking cessation counseling training for pediatric residents in the continuity clinic setting.

Reason for exclusion: This study was a quasi-experimental cross-over design with pre-test and post-test for each group. The groups were created using non-random methods.

Leong SL, Lewis PR, Curry WJ, Gingrich DL. Tobacco world: evaluation of a tobacco cessation training program for third-year medical students.

Reason for exclusion: This study was a cohort design using pre-test and post-test measures with comparison groups for a pilot year and for an intervention year. The authors state the pilot study year was experimental; however, the method of student assignment to groups was unclear.

Madlon-Kay DJ, Harper PG, Reif CJ. Health promotion counseling in residency training.

Reason for exclusion: This study reports a pre-test post-test non-experimental design with teaching faculty and resident groups.

McIlvain HE, Susman JL, Manners MA, David CM, Gilbert CS. Improving smoking cessation counseling by family practice residents.

Reason for exclusion: This study was a quasi-experimental design with resident pre- and post-training measures and no comparison group.

Molina AJ, Fernandez T, Fernandez D, Delgado M, de Abajo S, Martin V. Knowledge, attitudes and beliefs about tobacco use after an educative intervention in health sciences' students.

Reason for exclusion: This was a cohort study with a control group. The method of assigning intervention or control status was not stated.

Moushey E, Shomo A, Elder N, O'Dea C, Rahner D. Community partnered projects: residents engaging with community health centers to improve care.

Reason for exclusion: This study describes the development of a quality improvement initiative.

Pederson LL, Blumenthal DS, Dever A, McGrady G. A web-based smoking cessation and prevention curriculum for medical students: why, how, what, and what next.

Reason for exclusion: This was a cohort study with one comparison group and no control group.

Pignataro RM, Gurka M, Jones DL, Kershner RE, Ohtake PJ, Stauber W, et al. Educating physical therapist students in tobacco cessation counseling: Feasibility and preliminary outcomes.

Reason for exclusion: This study was a cohort study and two cohorts were compared with pre-test and post-test measures. The cohort groups existed and were not randomly created.

Ramseier CA, Burri M, Berres F, Davis JM. The implementation of a tobacco dependence education curriculum in a Swiss Dental Hygiene School – an 8-year review.

Reason for exclusion: This study was a retrospective descriptive program evaluation over eight years.

Rigotti NA. Training future physicians to deliver tobacco cessation treatment.

Reason for exclusion: This study was descriptive in nature and included a critique of a key study in medical education research.

Romito L, Budyn C, Oklak MA, Gotlib J, Eckert GJ. Tobacco use and health risks in two dental clinic populations: Implementation and evaluation of a brief targeted intervention.

Reason for exclusion: This study describes one cohort of students' post-intervention evaluation of smoking cessation counseling program content.

Roseby R, Marks MK, Conn J, Sawyer SM. Improving medical student performance in adolescent anti-smoking health promotion.

Reason for exclusion: This was a cohort study with post-training measures compared to a non-randomized student comparison group.

Schoonheim-Klein M, Gresnigt C, van der Velden U. Influence of dental education in motivational interviewing on the efficacy of interventions for smoking cessation.

Reason for exclusion: This study was a cohort design with three intervention groups and a control group.

Secker-Walker RH, Solomon LJ, Flynn BS, LePage SS, Crammond JE, Worden JK, et al. Training obstetric and family-practice residents to give smoking cessation advice during pre-natal care.

Reason for exclusion: This is a one-group before and after design with regard to residents. The residents were trained to deliver the control or intervention protocol to patients based upon the patient randomization procedure. This study was excluded based on study design.

Shershneva M, Kim JH, Kear C, Heyden R, Heyden N, Lee J, et al. Motivational interviewing workshop in a virtual world: learning as avatars.

Reason for exclusion: This study was a cohort design without a control group, using mixed methods to assess learning. The population was a combination of licensed physicians and residents.

Simansalam S, Brewster JM, Mohamed MHN. Training Malaysian pharmacy undergraduates with knowledge and skills on smoking cessation.

Reason for exclusion: This study was a cohort design with a comparison group and pre- and post-intervention measures.

Singleton JA, Carrico RM, Myers JA, Scott DA, Wilson RW, Worth CT. Tobacco cessation treatment education for dental students using standardized patients.

Reason for exclusion: This study was a cohort study with control group and pre- and post-intervention measures.

Soong JL, Wilken L. Efficacy and satisfaction of a smoking cessation telephone counseling service provided by pharmacy students.

Reason for exclusion: This study was a prospective research survey to evaluate smoking cessation counseling delivered by one student cohort.

Spangler J, Foley KL, Crandall S, Lane C, Walker K, MacRae M, et al. Implementing smokeless tobacco instruction into medical student education: addressing the gap.

Reason for exclusion: This study was a cohort design with pre-test and post-test measures and historical controls.

Strayer SM, Martindale JR, Pelletier SL, Rais S, Powell J, Schorling JB. Development and evaluation of an instrument for assessing brief behavioral change interventions.

Reason for exclusion***:*** This was a psychometric study of a coding instrument for standardized patient interviews.

von Garnier C, Meyer M, Leuppi J, Battegay E, Zeller A. Smoking cessation counselling: impact of chart stickers and resident training.

Reason for exclusion: This study was a cohort study with a historical pre-interventional cohort.

Wadland WC, WinklerPrins VJ, Noel MM, Thompson ME, Rios-Bedoya CF. Student performance on smoking cessation counseling with standardized patients.

Reason for exclusion: This study was a cohort design comparing two cohorts and no control group.

Waheedi M, Al-Tmimy AM, Enlund H. Preparedness for the smoking cessation role among health sciences students in Kuwait.

Reason for exclusion: This study was a cohort design with a comparison group. No student clinical performance counseling outcomes or patient outcomes were reported.

Willett LL, Palonen K, Allison JJ, Heudebert GR, Kiefe CI, Massie FS, et al. Differences in preventive health quality by residency year.

Reason for exclusion: This study was a cross-sectional design.

Supplementary Section S3. Grey literature studies excluded on full text read

POPULATION

Simon SE. Motivational interviewing for smoking cessation.

Reason for exclusion: In this dissertation the population included both nurse practitioner students and licensed nurse practitioners and the data were not presented separately. The study was a one-group pre-test post-test design and there was no control group.

COMPARATOR

Strecker BD. Implementation of a tobacco use intervention program into clinical dental hygiene education: A program evaluation.

Reason for exclusion: This master's thesis study was a cohort design with pre-test and post-test measures, and no control group.

Whitehead D. Tobacco cessation education for advanced practice nurses.

Reason for exclusion: This study was cohort design with advanced practice nursing students with pre-test and post-test measures and no comparison group.

STUDY DESIGN

Chalmers K, Seguire M, Brown J. Tobacco use and baccalaureate nursing students: a study of their attitudes, beliefs and personal behaviours.

Reason for exclusion: This study was a cross-sectional survey design.

Guiffre AM. Preparing medical students to counsel for smoking cessation.

Reason for exclusion: This dissertation study was a cross-sectional design comparing surveys of two cohorts of medical students.

Harris JL. Tobacco cessation education in North Carolina dental hygiene programs.

Reason for exclusion: This master's thesis study was a cross-sectional survey design.

Hatfield AS. Personal tobacco use behaviors and tobacco cessation activities of dental and dental hygiene students in United States dental schools.

Reason for exclusion: This master's thesis was a cross-sectional survey design.

McCartan B, McCreary C, Healy C**.** Attitudes of Irish dental, dental hygiene and dental nursing students and newly qualified practitioners to tobacco use cessation: a national survey.

Reason for exclusion: This study was a cross-sectional survey design.

Nims CL. A national study of OB/GYN residency directors: Smoking cessation education of residents.

Reason for exclusion: This dissertation study was a cross-sectional survey design.

Patelarou E, Vardavas CI, Ntzilepi P, Warren CW, Barbouni A, Kremastinou J, et al. Nursing education and beliefs towards tobacco cessation and control: a cross-sectional national survey (GHPSS) among nursing students in Greece.

Reason for exclusion: This study was a cross-sectional survey design.

Pignataro RM. The role of tobacco cessation counseling in physical therapist practice and education.

Reason for exclusion: This dissertation study consisted of three studies. Study 1 was a literature review. Study 2 was a cross-sectional survey design. Study 3 was a pre-test post-test design with two cohorts of student physical therapists at two university sites. There was no comparison group.

Rosselle TL. Nursing students' perception of their role in providing tobacco cessation education.

Reason for exclusion: This master's thesis study was a cross-sectional, descriptive survey design.

Tresolini CP. Medical students' development of self-efficacy in conducting patient education for health promotion: An analysis of learning experiences.

Reason for exclusion: This dissertation study was a qualitative case study design.

DUPLICATE OF PUBLISHED STUDIES

Cannick GF. Development, implementation, and evaluation of a standardized patient-based training program in oral cancer prevention and early detection for dental students.

Reason for exclusion: This dissertation research was a literature review and three publishable studies' design. In the second study, a RCT design using pre-test and post-test measures with intervention and control groups assessed student tobacco counseling skills using observers. The same RCT study was published by Cannick and colleagues in 2007 and the published version was selected for this systematic review.

Singleton JA. Tobacco dependence treatment education for dental students using standardized patients.

Reason for exclusion: This dissertation was a group randomized quantitative study design with pre-test and post-test measures. The same RCT study was published by Singleton and colleagues in 2014 and the published version was selected for this systematic review.

Supplementary Table 1. Assessment of quality for meta-analysis articles, 1990 – 2017, n=13

**Study**	Q1	Q2	Q3	Q4	Q5	Q6	Q7	Q8	Q9	Q10	**%**
Adkins [86]	Y	N	U	N	U	U	Y	Y	Y	Y	50%
Allen et al.[75]	Y	N	U	U	U	Y	Y	Y	Y	Y	60%
Brame et al.[87]	Y	N	U	Y	U	U	Y	Y	Y	Y	60%
Cannick et al.[76]	Y	N	U	N	U	Y	Y	Y	Y	Y	60%
Carpenter et al.[77]	U	U	U	N	Y	N	Y	Y	Y	Y	50%
Cornuz et al.[78]	Y	N	Y	Y	Y	Y	Y	N	Y	Y	80%
Hebert et al.[79]	Y	Y	Y	Y	Y	Y	Y	Y	U	Y	90%
Koerber et al.[80]	U	U	U	Y	Y	U	N	Y	Y	Y	50%
Ockene et al.[81]	U	Y	U	Y	U	Y	Y	Y	N	Y	60%
Ockene et al.[82]	Y	N	U	U	Y	Y	Y	Y	Y	Y	70%
Rapp et al.[83]	Y	U	Y	U	U	Y	N	Y	U	Y	50%
Steinemann et al.[84]	U	U	U	Y	Y	Y	Y	Y	U	Y	60%
Strecher et al.[85]	U	N	U	Y	Y	Y	Y	Y	U	Y	60%
**Total %**	61	15	23	54	54	69	85	92	61	100	**61.5%**

Y=Yes, N=No, U=Unclear; JBI critical appraisal checklist for randomised control/pseudo-randomised trials: [71] Q 1= Was the assignment to groups truly random? Q 2 = Were participants blinded to treatment allocation? Q 3 = Was allocation to treatment groups concealed from the allocator? Q 4 = Were the outcomes of people who withdrew described and included in the analysis? Q 5 = Were those assessing the outcomes blind to treatment allocation? Q 6 = Were

the control and treatment groups comparable at entry? Q 7 = Were groups treated identically other than for he named interventions? Q 8 = Were outcomes measured in the same way for all groups? Q 9 = Were outcomes measured in a reliable way? Q10 = Was appropriate statistical analysis used?

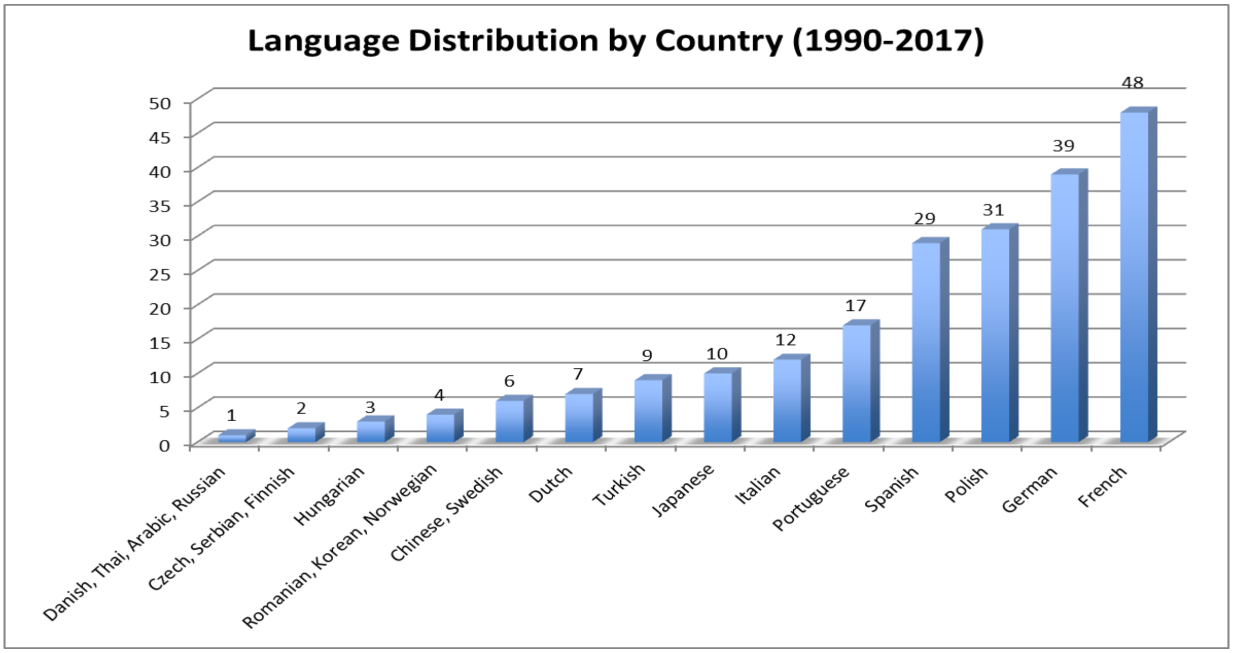

Supplementary Figure 2. Smoking cessation counseling publications, other than English, 1990-2017, N=239.

Supplementary Table 2. Theoretical underpinnings of RCT studies, 1990–2017, n=28

**Study**	5As CPGs [30,45]	Trans-theoretical [158,159]	MI Theory [160,161]	Self-efficacy Theory [155–157]	CPG	Other theories
Adkins [86]	X	X		X		Experiential Learning-Brookfield; Kolb
Allen et al.[75]	X					
Brame et al.[87]	X	X		X		Behavior modification interview techniques
Cannick et al.[76]	X					Precede-proceed theory [162]
Carpenter et al.[77]			X			
Cornuz et al.[78]	X	X				
Hebert et al.[79]				X		Patient centered counseling [81]
Koerber et al.[80]		X	X			
Ockene et al.[81]				X		Patient centered counseling [81]
Ockene et al.[82]	X			X		
Rapp et al.[83]	X	X				
Steinemann et al.[84]	X					
Strecher et al.[85]	X					
Non meta-analysis studies						
Carpenter et al.[91]		X				Facet based learning [163]
Coultas et al.[92]	X					

Garg et al.[93]					X	
Hymowitz et al.[94]	X	X	X			
Mounsey et al.[95]		X	X			
Papadakis et al.[96]					X	
Prochaska et al.[97]	X					
Roche et al.[98]	X					
Roche et al.[102]	X					Current research on smoking cessation intervention skills
Royce et al.[103]		X				Patient centered counseling[81] Individual & organizational change; Bandura[155-157]
Seim and Verhoye[99]				X		
Sejr and Osler[100]						Nursing education theoretical content
Stolz et al.[101]	X		X			
Strayer et al.[104]	X	X	X			
Ward and Sanson-Fisher[105]						Pendleton's rules; a consultation approach to teaching and learning

Supplementary Table 3. Student knowledge outcome summary table, n=9

<table>
<tr><th>Study</th><th>Measured by</th><th>Pre-Inter.</th><th>Post-Inter.</th><th>Comments</th></tr>
<tr><td>Adkins[86]</td><td>Knowledge of tobacco cessation counseling assessed by percent correct of 15 multiple choice questions involving four scenarios on stages of change, referrals and follow-up procedures, and available patient cessation resources.</td><td>Yes</td><td>Yes</td><td>
<table>
<tr><th></th><th>Pre-test n=31</th><th>Post n=27</th><th>Differ-ence ^</th></tr>
<tr><td colspan="4">Knowledge** Mean (SD)</td></tr>
<tr><td>Control n=15</td><td>67.9 (6.87)</td><td>72.8 (10.86)</td><td>4.9 (12.05)</td></tr>
<tr><td>Test n=12</td><td>72.1 (9.99)</td><td>78.4 (9.06)</td><td>6.4 (11.70)</td></tr>
</table>
**p=0.08 The mean post-test score showed no statistically significant difference between the 2 groups.

^ Difference between pre-test and post-test scores as Mean (SD) among dental hygiene students was 1.5.</td></tr>
<tr><td>Brame et al.[87]</td><td>Knowledge of tobacco cessation counseling assessed by percent correct of 15 multiple choice questions involving four scenarios on stages of change, referrals and follow-up procedures, and available tobacco dependence resources.</td><td>No</td><td>Some</td><td>Some knowledge scores on quit-line phone numbers and how to refer patients reported as percentages only at the six-month end of training date. No baseline or 6-month knowledge scores reported in the article with dental hygiene students.</td></tr>
<tr><td>Carpenter et al.[77]</td><td>Two 15-item multiple choice tests on brief negotiation (BN) and</td><td>No</td><td>Yes</td><td>No raw data reported. Groups of combined students: pharmacy, nursing, medicine, social work and dentistry. Coefficient alphas low</td></tr>
</table>

	tobacco cessation (QUITS) strategies.			(0.46 and 0.31). Scores on BN and QUITS were significantly correlated with standardized patient video assessment at post-test (r = 0.50, p <0.001; r = 0.55, p < 0.001, respectively).
Prochaska et al.[97]	Three multiple-choice items assessing students' tobacco treatment knowledge.	No	Yes	Overall knowledge scores did not differ by patient case version: 48% (SD=0.20) for the alcohol-positive case versus 40% (SD=0.21) for the alcohol-negative case, p=0.124. Greater knowledge was significantly correlated with tobacco treatment performance scores, r=0.17, p=0.034.
Rapp et al.[83]	Knowledge of diseases associated with smoking as responses to 9 diseases and summed to a score of medical knowledge.	Yes	Yes	Change between baseline & follow-up as nursing student increase in medical knowledge, N=949.<br>Int. group M= 0.97 (1.60)<br>Cont. group M = 0.53 (1.51)<br>Means ratio 1.59 (1.16; 2.17) p=0.0056
Roche et al.[102]	Smoking knowledge on a) morbidity and mortality associated with smoking; b) intervention strategies; c) intervention effectiveness; d) cessation practices assessed on by 32-item questionnaires.	Yes	Yes	Among undergraduate medical students at post-test, there were statistically significant (p=<0.05) increased knowledge scores among all intervention groups but not the control group.<br>Pre-test group M(SD) = 22.0(2.8)<br>Post-test group M(SD) = 23.6(1.88)

<table>
<tr><td>Steinemann et al.[84]</td><td>Knowledge about counseling techniques and smoking-related pathology was examined using a 26-point written quiz.</td><td>Yes</td><td>Yes</td><td>After smoking cessation education, surgical residents demonstrated a significant improvement about smoking pathology and smoking cessation resources, p<0.05.
<table>
<tr><td></td><td>Pre-
n=25</td><td>Post-
n=16</td><td>Differ-
ence ^</td></tr>
<tr><td colspan="4">Knowledge** Mean (SD)</td></tr>
<tr><td>EBM</td><td>19.9
(±2.8

n=13</td><td>20.3
(±2.8)

n=10</td><td>0.4</td></tr>
<tr><td>Role Play</td><td>20.6
(±2.3)

n=12</td><td>22.3
(±1.8)

n=6</td><td>1.7</td></tr>
</table>
</td></tr>
<tr><td>Stolz et al.[101]</td><td>20 item multiple-choice quiz for theoretical knowledge on the effect of smoking on health.</td><td>No</td><td>Yes</td><td>All 4 teaching methods provided medical students a similar level of knowledge: M(SD)
Int 1. 50.3(9.2); Int 2 47.4(8.8)
Int 3. 51.3(7.5); Int 4 47.8(8.5); (p=0.439)</td></tr>
<tr><td>Sejr and Osler[100]</td><td>Knowledge gain on smoking as a risk factor measured by 5 self-report statements scored 0-5.</td><td>Yes</td><td>Yes</td><td>Nursing student difference in knowledge (scored 0-25).
C 2.98(1.28) C 3.29(1.37)
I 2.91(1.59) I 3.67(1.38) p=0.04</td></tr>
</table>

Supplementary Table 4. Characteristics of meta-analyses studies, n=13

<table>
<tr><th>Study</th><th>Study Design</th><th>Participants</th><th>Intervention</th><th>Comparator</th><th>Outcomes / Notes</th></tr>
<tr>
<td>Adkins [86]

USA

Unpublished Master's thesis</td>
<td>2- parallel group randomized design
(Cluster-RCT)

Randomization:
Knowledge scores ranked subjects after pre-test.
To remove knowledge as a possible confounder for high confidence scores, the Test & Control groups were randomized using equal numbers of subjects scoring above & below median to assure each group was comprised of equal numbers of high & low scorers on knowledge.</td>
<td>Population/Sample Size:
36 senior Dental Hygiene students University of North Carolina at Chapel Hill
Sample = 31
16 (Int) & 15 (control)

Theories:
Experiential Learning- Brookfield; Kolb; (SPs); US Public Health Service Guideline on S/C Counseling; Prochaska & Di Clemente Stages of Change Model; Self-efficacy</td>
<td>All students received standard curriculum: TCC lecture, then "test" group randomly assigned to SP encounter of 15 minutes who was in "Contemplation" Stage of Change. SP provided written feedback on student performance.
At 6 days after SP training post-test given to both groups.
One week after post-test, all students debriefed to gather student perceptions of TCC lecture and SPs.
Duration: Two weeks</td>
<td>No SP training</td>
<td>Dental Hygiene outcomes: Continuous
Self-reported confidence measured by visual analogue scale (0-10) on 16 TCC-related tasks and knowledge of TCC measured by percent correct of 15 multiple choice questions on stages of change, referral and cessation resources.
<table>
<tr><th></th><th>Pre n=31</th><th>Post n=27</th><th>Total</th></tr>
<tr><td colspan="4">Confidence* Mean (SD) *p=0.02</td></tr>
<tr><td>Cont</td><td>5.9 (2.09)</td><td>7.0 (2.14)</td><td>15</td></tr>
<tr><td>Test</td><td>6.1 (1.38)</td><td>8.3 (1.17)</td><td>12</td></tr>
<tr><td colspan="4">Knowledge** Mean (SD) **p=0.08</td></tr>
<tr><td>Cont</td><td>67.9 (6.87)</td><td>72.8 (10.86)</td><td>15</td></tr>
<tr><td>Test</td><td>72.1 (9.99)</td><td>78.4 (9.06)</td><td>12</td></tr>
</table>
Patient outcomes: Not assessed.
Author conclusions: Students who had TCC lecture plus SP training experienced a statistically significant increase in overall self-reported confidence in their ability to deliver TCC compared to their classmates who had the lecture alone. A significant increase in overall knowledge was not found.</td>
</tr>
</table>

<table>
<tr><td>Allen et al.[75]

USA</td><td>RCT with blocking on type of clinic and year of residency of participating physicians.
Adherence to intervention protocol based on real patient exit interview
Patients randomized; measured by self-reported quit rates, saliva cotinine level</td><td>Population/Sample Size:
Family & internal medicine residents

Family medicine:
19 (Int); 14 (Cont.)
Internal medicine:
46 (Int.); 39(Cont.)
Total (n=118):
65 (Int.); 53 (Cont.)

African-American patients; (n=1086) Avg. age 44, avg. smoking 25 years, 14 cigs/day</td><td>One, 2-hour training session on health effects of smoking, benefits of quitting, withdrawal, relapse, smoking cessation, counseling; lectures, videotapes and rehearsal of brief (3-5) minute intervention
Duration: 26 months, telephone follow-up 3 - 12 months post-enrolment to assess current smoking behavior

Theories: 5As model</td><td>No training in smoking cessation, or SC materials</td><td>Resident physician outcomes: Advise to Quit Smoking.
- 70% of Int. patients & 16% in Cont. group were advised to quit

Patient outcomes:
Self-reported quit rates at 3 & 12 mo. were higher in control than intervention group, ns

Saliva cotinine level biochemically validated at
3 mo. Int. 2%-3.2%; & Cont. 1.8% -2.7%
12 mo. Int. 2.2%-3.7%; Cont.2.8%-4.6%</td></tr>
<tr><td>Brame et al.[87]

USA</td><td>2- parallel group randomized design
(Pilot study)
Randomization: Following pre-test, knowledge scores ranked subjects.
Knowledge at baseline of TCC measured by % correct of 15 Multiple-choice questions on stages of change, referrals and available TCC</td><td>Population/Sample Size:
Senior dental hygiene students University of North Carolina at Chapel Hill
Sample = 31
16 (Int) & 15 (control)</td><td>Description:
All students receive 3 hr of tobacco cessation education. One week after lecture all students -baseline evaluation.
At 6 days after SP training post-test to measure confidence of TCC. At 6 months post-training and prior to graduation, same measure of confidence used.
Duration: 6 months</td><td>No SP training</td><td>Dental Hygiene student outcomes: Continuous
Confidence measured by visual analogue scale (0-10)
Confidence scores
<table>
<tr><td></td><td>Mean (SD)</td><td>Total n</td><td>P values</td></tr>
<tr><td>Baseline</td><td></td><td></td><td></td></tr>
<tr><td>Cont</td><td>5.9 (2.1)</td><td>15</td><td></td></tr>
<tr><td>Test</td><td>6.1 (1.4)</td><td>16</td><td></td></tr>
</table></td></tr>
</table>

Brame et al.[87] (con't)	resources.<br>To remove knowledge as a possible confounder for high confidence scores, the Test & Control groups were randomized using equal numbers of subjects scoring above & below median to assure each group was comprised of equal numbers of high & low scorers on Knowledge.		**Theories**: Stages of Change Model; 5As and 5Rs counseling; Self-efficacy; behavior modification interview techniques; SPs		**End-training at 6 days and baseline** — *p*=0.002<br>**Cont** 6.9 (2.1) 14<br>**Test** 8.3 (1.2) 13<br>**6-Month follow-up between end of training & 6 mo** — *p*=0.09<br>**Cont** 8.1 (1.2) 13<br>**Test** 8.0 (1.3) 13<br><br>**Outcomes:** Student knowledge outcomes not reported and patient outcomes not assessed.<br>**Author conclusions:** SP training improved students' initial confidence in providing TCC and was sustained but not to a significant degree. Control group continued to gain confidence in the 6 months after training & at 6 months showed little difference and clinical experience may have increased confidence.
Cannick et al.[76]<br><br>USA	RCT- students randomized 3 weeks after OSCE & post-test OSCE at 6 mo. follow-up	**Population/Sample size**:<br>1st & 2nd year pre-doctoral dental students (n=104)	Usual curriculum + one 2-hr faculty facilitated session with SPs.<br>Dental faculty demonstrated how to counsel while doing head and neck exam; faculty observed student practice	No training session beyond normal course work	**Dental student outcomes: Continuous**<br>5As tobacco cessation counseling skills is the mean difference between pre-test and post-test scores, scored 0=not performed; 1 performed; total possible=5<br>**Patient outcomes**: not reported

Cannick et al.[76] (con't)	Tobacco cessation counseling skills (5As) measured by OSCE videotape of SP who smokes.	Int Control<br>Yr 1 23 21<br>Yr 2 24 28<br>Total n=96	**Duration:** Prior to study all students received tobacco cessation counseling techniques in a preventive dentistry course.<br>**Theories**:<br>Precede-proceed theory; CPG by Fiore		Pre Post Difference<br>**Yr 1**<br>Cont (n=21) 20.6(6.8) 21.9(4.2) (p=0.47)<br>Int (n=23) 23.2(3.8) 21.3(4.4) (p=0.09)<br>**Yr 2**<br>Cont (n=25) 23.4(5.1) 21.1(3.6) (p=0.05)<br>Int (n=24) 23.0(4.3) 22.2(4.5) (p=0.51)<br>**Author conclusions:** More intensive training than a "one shot" intervention is needed for tobacco dependence counseling
Carpenter et al.[77]<br>USA	RCT: Health care providers in training randomized to intervention or control. No follow-up beyond pre- post-test analysis<br>Measured by mean difference in brief tobacco cessation interventions grounded in the motivational interviewing with a SP who smoked.	**Population/Sample**<br>Pharmacy 28%; nursing 21%; medical 21%; social work 18%; dental 7.5%<br>(n=76 in both Int & Control groups)	**Description**: Two online tutorials of 2 hr total, basic principles of motivational interviewing (BN-brief negotiation) skills & brief tobacco cessation interventions (QUITS).<br>Interactive exercises & tailored feedback, interactive practice & review, video & audio examples, self-reflection.<br>**Theories**: Motivational interviewing-Miller & Rollnick	**Comparison** intervention:<br>Reading materials mirrored the content of the intervention.	**Health care students' outcomes:** Responses (scored 0 – 3; total =57) from expert review of pre- and post-test computerized standard patient video assessments. (SPVA).<br>Intervention 19.1 p<0.001<br>Control 13.5<br>**Pre-test** Min Max M SD<br>Tutor. 4.0 25.5 12.6 5.1<br>Read 2.0 26.0 14.5 5.4<br>**Post-test**<br>Tutor. 2.0 44.0 31.7 6.6<br>Read 9.0 43.5 28.0 6.1<br>**Student knowledge quizzes**:<br>Knowledge quizzes two 15-item multiple choice tests at post-test only; no baseline data and coefficient alpha low, not usable

Cornuz et al.[78]<br><br>Switzerland	RCT: cluster randomized<br><br>Residents and patients were randomized<br><br>Follow-up after patient hospital visit at 3 months to assess physician counseling practices (measured (after 3 visits) by real patients scored 0 - 14, and resident self-reported perceived confidence and effectiveness at 3-months (10 point scale); and<br><br>at one-year to assess patient smoking abstinence (measured by self-report & exhaled carbon monoxide (only 1 clinic)	**Population/Sample**<br><br>Internal or family medicine residents (n=35)<br><br>Int. n=17;<br><br>Cont. n=18<br><br>251 consecutive smokers at adult ambulatory clinics<br><br>n=115 (Int)<br><br>n=136 (Cont)	**Duration**:<br><br>Two half-day sessions on stage matched smoking cessation interventions, videotaped encounters, role-play, and practise with 4 SPs; written materials, active learning techniques.<br><br>**Theories**:<br><br>5As model, pharmacotherapy techniques, trans-theoretical model of change	**Comparison** intervention.<br><br>One half-day didactic training on screening, diagnosis & dyslipedemia management	**Resident physician outcomes**:<br>Interv. Control<br><br>Score for counseling M(SD) Total M(SD) Total<br>4.0(0.3) n=17 2.7(0.2) n=18<br>(p<0.001)<br><br>**Resident** Interv. Control<br><br>Score 3-mos. M Total M Total<br><br>confidence 7.7 n=17 5.2 n=18<br>(p<0.002)<br><br>effective 6.8 n=17 5.4 n=18<br>(p<0.09) * no baseline data for residents<br><br>**Patient outcomes: smoking abstinence**:<br>Inter. n=115 Cont. n=136<br><br>1 year 15 (13%) 7 (5%) (p<0.005)<br><br>**Author conclusions:** At one year, residents' frequency & quality of counseling improved & patients of trained residents showed significantly higher S/C rates than untrained residents

Hebert et al.[79]<br><br>USA	RCT-Patients randomized<br><br>In addition to counseling interventions (x3), patients were R assigned to physician and follow-up condition which was<br><br>**Maximal** follow-up =3 phone calls at 1,2, & 3 months by health counsellors<br><br>or **Minimal** =no further counseling calls (from Ockene et al.[81])	**Population/Sample size**:<br><br>Adult smokers (n=1286) were recruited over 3 years July 1985 to June 1988 seen by residents in 2 internal medicine and 3 family practice clinics [primary care settings]<br><br>N=196 residents:<br><br>Internal medicine (n=150) & family practice (n=46)	**Description**:<br><br>All residents had 2.5 hr training on all 3 conditions with slides, videotapes, and role play. Each resident was videotaped doing patient-centered counseling and received feedback from instructor<br><br>**Counseling** (**CI**) In addition to AO, a 5-10 intensive intervention, assessing motivation to quit, resources for change and developing a plan for cessation, plus community resources and arrange for a follow-up with resident in 1-2 weeks<br><br>**Theories**:<br><br>Cognitive & behavioral basis with focus on development of patient self-efficacy; (Bandura); current research; patient-centered counseling theory + feedback	**Counseling plus Gum with Nicotine (NCG)**<br><br>In addition to AO & CI, patients willing to set a quit date were given a Rx for NCG, and referred to clinic assistant on proper use of gum<br><br>**Advice only** (**AO**) =referent group<br><br>Advise to stop smoking; advice tailored to client heath issue & **if** patient interested provision of S/C resources & Nicotine gum	**Patient outcomes**: Smoking status at 6 months measured by patient report<br><br>**AO** / **CI** / **CI + NCG**<br>n %quit / n %quit / n %quit<br>Male: 152 11.2 / 136 18.4 / 123 23.6<br>Fem.: 208 11.1 / 198 11.6 / 180 20.6<br>*p*<0.10<br><br>**Author conclusions:**<br><br>CI + NCG had an overall pattern of greater effectiveness for both more and less addicted smokers, with the highest levels of cessation (about 27-30%) among less addicted smokers.

Koerber et al.[80]<br><br>USA	RCT; pilot study<br><br>Trained person rated counseling techniques in Brief MI (5-10 minutes) in counseling a SP to quit smoking using videotaped encounters<br><br>Student perceived efficacy in promoting patient behavior change in S/C as measured on Change Scale with 4 point rating scale (Max=16) by standardized patient (2 items) and student (2 items)	**Population/Sample Size**:<br><br>Junior & senior dental students<br><br>N=22; 11 in Int. and 11 in Control; all 22 students completed the study<br><br>All students had a prior 2-3 hour course on smoking cessation counseling (5As), oral health effects of tobacco in standard curriculum	**Description**:<br><br>12 hr. of Brief MI of 3 sessions of 4 hr. each at one week intervals (over 1 month) by licensed psychologist<br><br>#1 Basic principles of MI; nature of motivation; Basic counseling skills; reduce resistance; negotiate change #2 Establish rapport; set an agenda; explore readiness to change #3 Do brief MI with exercises to apply above content<br><br>**Theories**: Motivational Interviewing by Miller & Rollnick; Stages of Change Model	No training in Brief MI	**Dental student outcomes**: Mean # of S/C behaviors<br><br>**Int:** **Control**<br><br>Baseline 1.6 (0.9) 1.8(1.5)<br><br>Final 2.9 (1.3) * 1.3(1.0)<br><br>*Significant in MANOVA<br><br>**Student** perceived efficacy in promoting patient behavior change in S/C<br><br>**Int:** **Control**<br><br>Baseline 9.5(2.3) 9.4(1.3)<br><br>Final 10.0(2.1) 9.6(2.2)<br><br>*Not Significant in MANOVA<br><br>**Author conclusions:** Trained students were more likely to use BMI techniques; we did not find support for rapport, perceived efficacy, or confidence/likelihood to do SC counseling
Ockene et al.[81]<br><br>USA	RCT-Patients randomized<br><br>In addition to counseling interventions (x3) patients were R assigned to physician and follow-up condition	**Population/Sample size**: 196: Internal medicine (n=150) & family practice (n=46) residents	**Description**:<br><br>All residents had 2.5 hr. training on all 3 conditions- with slides, videotapes, and role-play Each resident was videotaped doing patient-centered counseling and received feedback from instructor.	**Counseling plus Gum with Nicotine (NCG)**<br><br>In addition to AO & CI, patients willing to set a quit date were given a Rx for NCG, and	**Resident physician outcomes**: 5As compliance measured by patients at time of clinic intervention<br><br>99% agreed residents had discussed the patient's smoking status;<br><br>91% agreed that advice to quit smoking had been provided;<br><br>85% agreement in the setting of a quit date;<br><br>88% agreed that some form of written materials about smoking had been handed out and received;

Ockene et al.[81] (con't)	which was **Maximal** follow-up =3 phone calls at 1,2, & 3 months by health counsellors or **Minimal** =no further counseling calls<br><br>Medical resident adherence to S/C protocol following the 5As measured by 249 randomly selected patients and their physicians at the time of initial intervention	Adult smokers (n=1286) were recruited over 3 years July 1985 to June 1988 seen by residents in 2 internal medicine and 3 family practice clinics [primary care settings]	**Counseling** (**CI**) In addition to AO, a 5-10 intensive intervention, assessing motivation to quit, resources for change and developing a plan for cessation, plus community resources and arrange for a follow-up with resident in 1-2 weeks<br><br>**Theories**:<br><br>Cognitive & behavioral basis with focus on development of patient self-efficacy; (Bandura); current research; patient-centered counseling theory + feedback	referred to clinic assistant on proper use of gum<br><br>**Advice only** (**AO**) =referent group<br><br>Advise to stop smoking; advice tailored to client heath issue & **if** patient interested<br><br>provision of S/C resources & Nicotine gum	90% agreed that NCG had been offered or a prescription written for its usage.<br><br>Patients reported that 9.4 minutes on providing advice to stop smoking, with averages of 14.7 and 15.1 minutes spent on CI and CI + NCG, respectively.<br><br>Residents' average reports of time spent were lower: 6.7, 12.3, and 13.9 minutes for AO, CI, and CI + NCG.<br><br>**Author conclusions:** Patients receiving brief patient-centered behavioral interventions with or without NCG, were much more likely to change their behaviors than those who received the AO intervention. Follow-up counseling by ancillary staff did not independently contribute to cessation.
Ockene et al.[82]<br><br>USA	RCT-10 school randomized trial<br><br>1. Student tobacco treatment skills using the 5As and pharmacotherapy counseling measured by OSCE scored on 33 item checklist by trained raters of videotaped OSCEs (Year 3)	**Population/Sample size**:<br><br>Medical students: 1096 in OSCE analysis:<br><br>TE (n=531) & MME (n=565).<br><br>Self-efficacy analysis (n=1047):<br><br>TE (n=495) & MME (n=552)	**Description**:<br><br>**MME:** All students had 4 hr self-paced Web-based course covering 5As with attention to Assist and Arrange pharmacotherapy counseling<br><br>2) role-play classroom demonstration and practice during Web course in first or third year with clerkship preceptors who had	**Comparison**<br><br>**TE**: largely didactic tobacco treatment interspersed among basic sciences and behavioral counseling classes in Years 1 and 2 of medical school. Some	**Medical students outcomes**:<br><br>**OSCE outcomes:** MME students completed more tobacco counseling behaviors on the OSCE checklist (mean 8.7 [SE 0.6] vs. mean 8.0 [SE 0.6], *p*=0.52 than TE students. No significant difference in total OSCE scores (*p*=0.41).<br><br>**Student self-efficacy in tobacco treatment outcomes.** A higher % of MME than TE students at post-intervention reported they were able to advise and assess (*p*<0.10) & assist, arrange and provide pharmacotherapy counseling (*p*<0.05)

<table>
<tr>
<td>Ockene et al.[82] (con’t)</td>
<td>2. Student self-reported self-efficacy skills for performing 5As and pharmacotherapy counseling (6 items in total)</td>
<td></td>
<td>been trained in tobacco dependence guidelines, and how to role model observe, instruct and give constructive feedback to medical students; 3) clerkship booster session in Year 3 family or internal medicine rotation.

Theories: Cognitive & behavioral basis with focus on development of student self-efficacy (Bandura); current & previous research; 5As counseling theory</td>
<td>schools included tobacco case studies as part of their behavioral counseling

(TE) was < 4 hours over the 4 years and varied in content</td>
<td>Author conclusions: Educational models need to include core knowledge and role-play in Yr. 1 and 2, more intensive follow-up for medical school preceptors and encounters with at least 2-3 smokers in clinical settings. The significant change in self-efficacy will likely result in eventual change in behavior.</td>
</tr>
<tr>
<td>Rapp et al.[83]

Germany</td>
<td>RCT-32 of 40 nursing schools Cluster Randomized

Knowledge of diseases associated with smoking was measured by responses to 9 diseases and summed to a score of medical knowledge

Students’ competence and knowledge in giving advice to people</td>
<td>Population/Sample size:
Year 1 & 2 nursing students

Baseline Follow-Up
Int 605 502
Cont 629 454

Total 1234 956</td>
<td>Description:
3-one day classes on how to give people advice to quit smoking given by trained teachers from participating schools using practice guidelines on brief SC interventions; focus on social, psychological, physiological aspects of smoking

Students role played counseling to smokers</td>
<td>Usual curriculum may have had traditional lessons about smoking</td>
<td>Nursing students’ outcomes: Change between baseline & follow-up
<table>
<tr><td></td><td colspan="2">Interven.</td><td colspan="2">Control</td></tr>
<tr><td></td><td>M</td><td>SD</td><td>M</td><td>SD</td></tr>
<tr><td>Increase in medical knowledge
p=0.0056</td><td>0.97</td><td>1.60</td><td>0.53</td><td>1.51</td></tr>
<tr><td>Compe-tence to advise people to quit smoking
p<0.0001</td><td>0.46</td><td>1.00</td><td>0.048</td><td>0.95</td></tr>
</table>
</td>
</tr>
</table>

Rapp et al.[83]<br>(con't)	who want to quit smoking measured with 2 statements and responses coded into 4 categories: strongly agree; agree to some extent; disagree to some extent; and strongly disagree.	**Duration**: Baseline and F/Up self-administered surveys. F/Up was on average 13.2 mo.	**Theories**: Stages of Change; CPGs by Fiore		Students' self-perceived competence to give advice to people who want to quit smoking improved with no change in control group (Means ratio [Int *vs*.Cont.] MR=1.51, 95% CI 1.29-1.78)<br><br>**Author conclusions:** The educational program was successful in delivering medical knowledge about S/C counseling and in enhancing nursing students' self-perceived knowledge in giving advice to smokers
Steinemann et al.[84]<br><br>USA	RCT<br><br>Smoking cessation counseling skills were evaluated by SPs portraying smokers with smoking related disease using checklists based on the 5As of smoking cessation counseling	**Population/Sample size**:<br>Surgical residents (N=25)<br><br>**Int**-N=13 Int 2 N=12<br>EBM Role-play<br><br>Pre 13 Pre-12<br>Post 10 Post 06<br><br>No control group	**Description**:<br><br>**Int 1** (EBM) 2hr of instruction:<br>1 hour round table discussion after receiving articles (1 hr reading) on tobacco pathophysiology and S/C counseling techniques<br><br>**Theories**: Current medical research on teaching residents; CPGs	**Int 2** (Role-Play)<br>2 hours of instruction: a 1-hour lecture on S/C, immediately followed by 1 hour of role playing exercises. Peer & faculty feedback (4 faculty hours) on performance of counseling skills after portraying a smoking patient or physician providing S/C counselling	**Resident outcomes:**<br><br>EBM **Int 1** Role-Play **Int 2**<br>Base Post Base Post<br>S/C skills 4.6(2.5) 8.8 4.1(3.3) 9.1<br><br>**Author conclusions:** After either form of brief education, residents demonstrated significant improvements in knowledge, attitude and skills in smoking cessation counseling.<br>Note: no group means reported for knowledge gains, or attitudes toward counseling.

Strecher et al.[85]<br><br>USA	RCT: factorial design: 4 groups<br><br>Resident counseling frequency, content, & attitudes were measured by self-report and by patient exit interviews (aggregated by physicians)<br><br>Research assistants interviewed patients right after visit with structured questionnaire<br><br>Patient quit rates were measured by patient report at 6 months after interview by phone; patient quit rates were biochemically verified	**Population/Sample size**:<br><br>261 residents from 3 postgraduate specialties-internal medicine, family medicine and pediatrics<br><br>**Duration**: All residents were taught minimal contact S/C counseling by a tutorial or a prompt or in combination, or none and assessed at 6 months (N=234) via post-test.<br><br>Patients (N=843) at outset & (N=659) at 6 month follow-up	**Description**:<br><br>**Tutorial**<br><br>2 sessions in S/C counseling: 1 hour group session onsite by faculty member, researcher, or clinical director who were trained in S/C teaching; 10 minutes on smoking and cessation, minimal contact approach with flowsheet; 2 videos of a session with a motivated & unmotivated patient; 20 minute group discussion<br><br>2 weeks later residents had a group/or individual session to discuss their S/C experience using checklists they were to complete on 3 patients	**Comparison**:<br><br>**Prompt** was identical to Tutorial.<br><br>Chart based reminders given to guide residents to counsel in SC.<br><br>Onsite nurses attached prompt sheet to patient/ parent charts of current smokers<br><br>**Control:** no intervention	**Resident numbers (n) and outcomes**:<br><br>**Resi-dents** | Tut +Pro | Tut | Prom | Control<br>**Pre** (n) | 66 | 66 | 60 | 58<br>**Post**(n) | 62 | 63 | 55 | 54<br><br>**Summary**: Residents receiving the tutorial reported a significantly higher post-test mean frequency than did non-tutorial residents (76% vs. 69%, *p*<0.05). Counseling frequency was higher for those who received the prompt than those who did not (75% vs. 70%, *ns*).<br><br>The mean number of S/C techniques reported by tutorial residents was double that reported by non-tutorial residents (1.5 vs 0.7, *p*<0.001). The number of techniques reported by those receiving the prompt was only slightly higher than those who did not (1.2 vs 1.0, *ns*).<br><br>Resident Attitudes: Only the tutorial produced significant changes in residents' attitudes. Those who received the tutorial significantly more often reported feeling more confident (72% vs. 48%) and successful (55% vs. 34%) than those not receiving the tutorial (*p*<0.001)

Strecher et al.[85] (con't)			**Theories**: Minimal contact smoking cessation counseling based on Strecher & other researchers, the 5As approach; behavior change theory		**Patient numbers and outcome:**<br><br>**Pre** (n) 250 234 228 225<br>**Quit**<br>**Rate**(%) 5.6 3.4 5.7 1.7<br><br>**Post**(n) 184 156 162 157<br>**Quit**<br>**Rate**(%) 8.2 5.3 6.3 5.2 (p>0.05)<br><br>**Summary**: Patient quit rates with residents in 3 intervention groups were higher than those in the control group (*ns*)<br>**Author conclusions:** To counsel effectively, physicians need training beginning in residency. Residents found tutorial more helpful than prompt. The intervention was feasible and effective in primary care residency training & significantly increased residents' self-reported S/C counseling practices and attitudes. Pediatric residents' responses to interventions did not differ from family or internal medicine.

Supplementary Table 5. Entry-level tobacco dependence education compared to usual or no educational program in health professional student training to promote client health

Patient or population: health professional student training to promote client health

Setting: Post-secondary educational institutions, colleges or universities, including undergraduate or pre-licensure training, postgraduate medical training and advanced practice nursing programs at the master's level

Intervention: Entry-level tobacco dependence education

Comparison: usual or no educational program

Outcomes	№ of participants (studies) Follow-up	Certainty of the evidence (GRADE)	Relative effect (95% CI)	Anticipated absolute effects	
				Risk with usual or no educational program	Risk difference with Entry-level tobacco dependence education
Change in number of 5 A's tobacco cessation counseling skills with standardized or real patients	1255 (4 RCTs)	⨁⨁◯◯ LOW [a]	-	-	SMD **1.03 SD higher** (0.07 higher to 1.98 higher)
Change in tobacco cessation counseling skills with standardized patients using a motivational interviewing approach	174 (2 RCTs)	⨁⨁⨁◯ MODERATE [b]	-	-	SMD **0.9 SD higher** (0.59 higher to 1.21 higher)
Patient smoking cessation at 6 months after counseling	1615 (3 RCTs)	⨁⨁⨁◯ MODERATE [c]	**OR 2.02** (1.49 to 2.74)	93 per 1,000	**78 more per 1,000** (39 more to 126 more)

Supplementary Table 5. Entry-level tobacco dependence education compared to usual or no educational program in health professional student training to promote client health

Patient or population: health professional student training to promote client health

Setting: Post-secondary educational institutions, colleges or universities, including undergraduate or pre-licensure training, postgraduate medical training and advanced practice nursing programs at the master's level

Intervention: Entry-level tobacco dependence education

Comparison: usual or no educational program

Outcomes	№ of participants (studies) Follow-up	Certainty of the evidence (GRADE)	Relative effect (95% CI)	Anticipated absolute effects	
				Risk with usual or no educational program	Risk difference with Entry-level tobacco dependence education
Patient smoking cessation at 1 year after counseling	1507 (3 RCTs)	⊕⊕◯◯ LOW [d]	**OR 1.04** (0.54 to 2.01)	101 per 1,000	**4 more per 1,000** (44 fewer to 83 more)
Change in students' self-efficacy in tobacco cessation counseling	1031 (4 RCTs)	⊕⊕◯◯ LOW [e]	-	-	SMD **0.38 SD higher** (0.11 lower to 0.87 higher)

***The risk in the intervention group** (and its 95% confidence interval) is based on the assumed risk in the comparison group and the **relative effect** of the intervention (and its 95% CI). **CI:** Confidence interval; **SMD:** Standardized mean difference; **OR:** Odds ratio

Supplementary Table 5. Entry-level tobacco dependence education compared to usual or no educational program in health professional student training to promote client health

Patient or population: health professional student training to promote client health

Setting: Post-secondary educational institutions, colleges or universities, including undergraduate or pre-licensure training, postgraduate medical training and advanced practice nursing programs at the master's level

Intervention: Entry-level tobacco dependence education

Comparison: usual or no educational program

Outcomes	№ of participants (studies) Follow-up	Certainty of the evidence (GRADE)	Relative effect (95% CI)	Anticipated absolute effects	
				Risk with usual or no educational program	Risk difference with Entry-level tobacco dependence education

GRADE Working Group grades of evidence
High certainty: We are very confident that the true effect lies close to that of the estimate of the effect
Moderate certainty: We are moderately confident in the effect estimate: The true effect is likely to be close to the estimate of the effect, but there is a possibility that it is substantially different
Low certainty: Our confidence in the effect estimate is limited: The true effect may be substantially different from the estimate of the effect
Very low certainty: We have very little confidence in the effect estimate: The true effect is likely to be substantially different from the estimate of effect

Explanations

a. I^2 = 94%; Participants not blinded to treatment allocation in one study; high risk of attrition bias in three studies; b. I^2 = 0%; Unclear documentation for randomization, allocation concealment, blinding of participants; c. I^2 = 0%; High risk of attrition bias in one study, plus imprecision in smoking cessation measured by self-report; d. I^2 = 69%; High risk of attrition bias in two studies; high risk of selection bias in one study; e. I^2 = 62%; High risk of attrition bias in one study
